# Albendazole induces immunotherapy response by facilitating ubiquitin-mediated PD-L1 degradation

**DOI:** 10.1136/jitc-2021-003819

**Published:** 2022-05-16

**Authors:** Lin Zhu, Xinwei Kuang, Guanxiong Zhang, Long Liang, Dandan Liu, Bin Hu, Zuozhong Xie, Hui Li, Hong Liu, Mao Ye, Xiang Chen, Jing Liu

**Affiliations:** 1 Department of Dermatology, Hunan Key Laboratory of Skin Cancer and Psoriasis, Hunan Engineering Research Center of Skin Health and Disease, Xiangya Clinical Research Center for Cancer Immunotherapy, Xiangya Hospital, Central South University, Changsha, Hunan, China; 2 Molecular Biology Research Center, Center for Medical Genetics, Hunan Province Key Laboratory of Basic and Applied Hematology, School of Life Sciences, Central South University, Changsha, Hunan, China; 3 Department of Research and Development, Beijing GAP Biotechnology Co., Ltd, Beijing, China; 4 Molecular Science and Biomedicine Laboratory, State Key Laboratory for Chemo/Biosensing and Chemometrics, College of Biology, College of Chemistry and Chemical Engineering, Collaborative Innovation Center for Chemistry and Molecular Medicine, Hunan University, Changsha, Hunan, China; 5 Research Center of Molecular Metabolomics, Xiangya Clinical Research Center for Cancer Immunotherapy, Central South University, Changsha, Hunan, China

**Keywords:** Melanoma, Drug Therapy, Combination, Immunotherapy, Tumor Escape

## Abstract

**Background:**

Immune checkpoint inhibitors (ICIs) have been increasingly used in patients with various cancers and have shown efficient therapeutic outcomes. However, fewer than 40% of cases across multiple cancer types show a response to ICIs. Therefore, developing more efficient combinational approaches with ICIs and revealing the underlying mechanisms are important goals for achieving rapid clinical transformation and application.

**Methods:**

The effects on antitumor immunity activity of albendazole (ABZ) and the synergistic effects of ABZ with CD73 blockade were investigated in the melanoma B16F10 and the Lewis lung cancer tumor-bearing immune-competent mice models. The mechanism of ABZ reducing PD-L1 protein level through suppressing UBQLN4 was identified and validated through immunoprecipitation-mass spectrometry and molecular methods. Bioinformatics and anti-PD-1 therapy melanoma patients samples analysis were used to assess the level of UBQLN4/PD-L1 in the therapeutic efficacy of anti-PD-1 therapy.

**Results:**

ABZ induces CD8^+^ T cell activity and subsequent immunotherapy response associated with suppression of PD-L1 protein level. Mechanistically, we revealed that ABZ promotes ubiquitin-mediated degradation of PD-L1 via suppressing UBQLN4, which was bound to PD-L1 and stabilized PD-L1 protein. Preclinically, genetic deletion or target inhibition of CD73 showed synergistic effects with ABZ treatment in the immune-competent mice models. Significantly, UBQLN4 and PD-L1 levels were higher in the tumor region of responders versus non-responders and correlated with better progression-free survival and overall survival in anti-PD-1 therapy melanoma patients.

**Conclusions:**

Our findings revealed a previously unappreciated role of ABZ in antitumor immunity by inducing ubiquitin-mediated PD-L1 protein degradation, identified predictors for assessing the therapeutic efficacy of anti-PD-1 therapy, and provided novel therapeutic possibility by combination treatment of ABZ and CD73 blockade in cancers.

## Introduction

Immune checkpoint inhibitors (ICIs) have been increasingly used in patients with various cancers, including metastatic melanoma and non-small-cell lung cancer (NSCLC), and have shown promising therapeutic outcomes.^1 2^ However, fewer than 40% of cases across multiple cancer types show a response to ICIs.^3^ Recent studies have demonstrated that ICIs combined with other treatment strategies induce rapid and substantial tumor regression. For instance, nivolumab, a specific anti-programmed cell death protein-1 (PD-1) antibody, combined with ipilimumab induced tumor regression in ~50% of metastatic melanoma patients, with 85% of patients surviving even after 1 year of treatment.^4^ Moreover, nivolumab is being investigated in combination with chemotherapies, immunotherapies, and vaccine-based therapies such as dendritic cell vaccines, NY-ESO-1 vaccines, and Tri-Mix in advanced melanoma patients.^5 6^ Therefore, finding novel combinatorial approaches with ICIs may benefit patients with cancer and hold a great deal of promise in this new era of cancer immunotherapies.

Drug repositioning and repurposing is an alternative strategy to discover and develop novel combined immunotherapy regimens. By reason of the tumor PD-L1 level is a determinant and a common biomarker for the assessment of the clinical response to anti-PD-L1/PD-1 therapy,^7^ we screened and found that albendazole (ABZ) could significantly reduce the expression of tumor cell membrane PD-L1 levels. ABZ is an FDA-approved broad-spectrum antiparasitic agent with low toxicity and is widely used in humans and animals.^8^ Recently, ABZ has been reported to possess antitumor activity in several cancer cell types,^9 10^ and one phase I clinical trial has been performed to determine the maximum tolerated dose of oral ABZ in patients with advanced cancer.^11^ Moreover, ABZ can enhance immunological responses; for example, ABZ increased the counts of CD4^+^ and CD8^+^ T cells and significantly stimulated the IFN-gamma (a Th1-type cytokine) response in mice and human patients infected with Echinococcus.^12–14^ However, the functions and underlying mechanism of ABZ in anti-tumor immunity remain unclear.

CD73 is a checkpoint molecule expressed on Treg cells and plays an important role in tumor immune escape, and is expected to be a next-generation target in immuno-oncology.^15^ With accumulating evidence implicating CD73 involved in cancer progression and immune escape, blockade of CD73 may be a potent anticancer therapeutic approach.^16^ For example, an anti-CD73 monoclonal antibody (mAb) significantly increased the antitumor effects of anti-CTLA-4 and anti-PD-1 mAbs in colon, prostate, and breast cancer models,^17^ and the combination of a CD73-specific inhibitor (adenosine 5'-(α,β-methylene) diphosphate, APCP) and an anti-CTLA-4 mAb synergistically suppressed melanoma tumor growth.^18^

In this study, we found that ABZ induces immunotherapy response, and promotes ubiquitin-mediated degradation of PD-L1 via suppressing UBQLN4, which is bound to PD-L1 and stabilized PD-L1 protein. We further evaluated the synergistic efficacy of ABZ in combination with CD73 blockages which is a potential strategy of combination immunotherapy for cancer treatment.

## Methods

### Cell culture

The human malignant melanoma cancer cell lines A375 and SK-MEL-28, Mouse melanoma cell line B16F10 and Lewis lung cancer (LLC) cells were purchased from American Type Culture Collection (ATCC). The human NSCLC cell lines A549, H460 and cell lines HEK293T come from our lab. Cells (A375, SK-MEL-28 and A549) were incubated in Dulbecco’s modified Eagle’s medium (DMEM, gibco, USA), others were cultured in RPMI1640 medium with 10% Fetal bovine serum (FBS, gibco, USA) and antibiotics (gibco, USA) at 37°C and 5% CO_2_ atmosphere. Test for mycoplasma contamination was performed routinely and all cell lines were verified to be negative. A375, SK-MEL-28 and A549 were pretreated with Interferon gamma (IFN-γ, 200 ng/mL) and subjected to a complete medium with the indicated concentration of ABZ dissolved in dimethyl sulfoxide (DMSO). Cycloheximide (200 ng/mL) was added for the indicated times. MG132 (10 µM) or Bafilomycin A1 (Baf A1, 100 nM) were then added to a complete medium 6–12 hours before the cell lines were harvested. The source and identifier of the aforementioned reagents were listed in online supplemental table 1.

10.1136/jitc-2021-003819.supp2 Supplementary data

### Clinical tissue samples

Paraffin sections of tumor tissue from melanoma patients treated with PD-1 mAb were collected from Xiangya Hospital. All tissue samples were collected in compliance with the informed consent policy. Clinical information is summarized in online supplemental table 2.

### Animal models

All in vivo experiments were conducted in strict accordance with the approval of the Animal Care and Use Committee of the Central South University (Changsha, Hunan, China, license no. 2019sydw011). Wild-type B16F10 cells (1×10^6^) or LLC cells (1×10^6^) were inoculated subcutaneously into 6-week-old Wide type or CD73 Knockout C57BL/6 female mice (from the shanghai SLAC). Nearly 1 week after inoculation, mice were randomly divided into designated experimental and control groups treated daily with ABZ (50 mg/kg/2 day i.p) and DMSO, respectively. In the other part, the combination treatment effect of ABZ and APCP was also observed. APCP (400 µg/mouse) was administered to the mice by the peritumoral (p.t.) on day 4 and day 8, combined therapy with ABZ (50 mg/kg/2 day i.p). At the endpoint, tumors were harvested and analyzed by fluorescence-activated cell sorting (FACS). The removed xenografts were also snap-frozen in liquid nitrogen.

For biochemical analysis of the blood routine examination of mice, taking a blood sample from the retro-orbital venous plexus and collecting it into anticoagulation tubes. The blood samples were investigated for routine complete blood count (CBC, XN-1000-B1) analysis including white cell count, white cell classification count, red cell count, hemoglobin, and platelets), neutrophil count, neutrophil ratio, lymphocyte count and lymphocyte ratio. H&E-staining of the kidney, liver, and spleen tissue were performed and analyzed by Hunan AiFang biological.

### FACS analysis of tumor immune cell

Single-cell suspension from mice B16F10 or LLC-xenograft tumor was obtained by rapid and gentle excising, physical grinding, and filter filtration. After blocking with trustain fcX anti-mouse CD16/32 (101320) antibody and getting rid of dead cells with Zombie Aqua Fixable Viability Kit (423102), enriched cells were stained using APC/CY7-CD45 (103116), CD3 (BV711-100241, APC-100236), PerCP/CY5.5-CD4 (100434), PE/CY7-CD8 (100722), APC-CD25 (17-0251-82), BV421-PD-1 (135218), BV605-CTLA4 (369610), PD-L1 (BV421-124315, APC-124312) for 25 min. After fixation and permeabilization (421402), intracellular GZMB was stained using GZMB (APC-372204, PE/Dazzle594-372216) antibody. Intranuclear FOXP3 was stained using PE/CY7-FOXP3 (25-5773-82) antibody. To detect the expression of PD-L1 on the membrane of single-cell suspension from human cell lines in culture, a PD-L1 (PE-329706) antibody is used. Cells were incubated with Human TruStain FcX (422302) block, and dead cells were gotten rid of with Zombie Aqua Fixable Viability Kit. The source and identifier of the aforementioned antibodies were listed in online supplemental table 1. Stained cells were analyzed by FACS Dxp AthenaTM and Aurora (Cytek, USA). Furthermore, data were processed by Flow Jo V.10.0 software.

### T cell-mediated tumor cell killing assay

T cell killing assay was performed as we previously described.^19^ To acquire activated T cells, healthy human donors peripheral blood mononuclear cells were isolated from the whole blood and cultured in CTSTM AIIM VTM SFM (A3021002; Gibco) with Immuno Cult Human CD3/CD28/CD2 T cell activator (10970; STEMCELL Technologies) and IL-2 (1000 U/mL, 202–1 L-050, R&D) for 1 week according to the manufacturer’s protocol. Once adhering to the plates, cancer cells were then treated with IFN-γ for 24 hours, incubated with ABZ (0, 0.625 or 1.25 µM) for 24 hours, then processed with activated T cells for 24 hours. The ratio between cancer cells and activated T cells is 1–3. T cells and cancer cell debris were removed through PBS buffer, and the rest of the cells were quantified by crystal violet staining.

The 3×10^5^ T cells were seeded in the 12-well plate. T cells were then treated with ABZ (0, 0.625, 1.25 µM) for 24 hours, centrifuge 300 g for 5 min to remove the medium, and the fixed cells were washed with PBS. Cells were incubated with Human TruStain FcX (422302) block, and dead cells were gotten rid of with Zombie Aqua Fixable Viability Kit. Enriched cells were stained using APC/Cya7-CD3 (300317), PE-CD8 (980902) and BV650-PD-1 (367429) antibodies at room temperature for 25 min. Stained cells were analyzed by FACS Dxp AthenaTM and Aurora (Cytek, USA). Furthermore, data were processed by Flow Jo V.10.0 software.

### RNA isolation, quantitative real-time PCR

Total RNA was extracted from cultured human cancer cells with TRIzol according to the standard protocol. cDNA was generated by SuperScript III First-Strand cDNA synthesis system with 500 ng total RNA. Quantitative PCR was performed using a qPCR system (Eppendorf, Hamburg, Germany) in accordance with the manufacturer’s instructions. The sequences of human PD-L1 and GAPDH primers were listed in online supplemental table 1. All mRNA expression levels were calculated using the comparative Ct method.

### Western blotting analysis

Western blotting was performed as we previously described.^20^ In brief, 30 µg content of total protein was subjected to SDS-polyacrylamide gel and subsequently transferred onto nitrocellulose membranes. After blocking with 5% skim milk resolved in TBST (Tris-buffered saline and Tween-20) for 1 hour, the membranes were then incubated with primary antibodies against PD-L1, UBQLN4, GAPDH, Ubiquitin, HA-Tag and His-Tag antibody, overnight at 4°C to detect target proteins. The source and identifier of the aforementioned antibodies were listed in online supplemental table 1. After incubation with a Peroxidase AffiniPure Goat Anti-Mouse IgG (H+L) or Peroxidase AffiniPure Goat Anti-Rabbit IgG (H+L) for 2 hours at room temperature. The protein blot was visualized using a chemical chemiluminescence imaging system. ImageLab (Bio-Rad, California, USA) was applied to process images.

### Co-immunoprecipitation

Endogenous co-immunoprecipitation (co-IP) was performed as we previously described.^21^ In brief, cells were lysed in a cold IP Lysis buffer containing protease inhibitor cocktail, in addition to 5% cell extracts saved as the input, the rest of protein lysates were incubated with primary antibody overnight at 4°C and then protein A/G agarose beads for 4 hour at 4°C. After overnight incubation, the immunocomplexes were washed three times with PBST (pH7.4 PBS with 0.1% Tirton-X100). Bound proteins were eluted by boiling with 2×SDS loading buffer before being resolved by SDS-PAGE.

### Pull-down assay

A375 cells with NC or HA-UBQLN4 overexpression were lysed using IP lysis buffer supplemented with protease inhibitor cocktail. Protein lysis was spun at 13,800 rpm for 15 min at 4°C and the supernatant was transferred to a new Eppendorf tube for use. After measuring the protein concentration, equal amounts of protein were extracted to incubate with Anti-HA magnetic beads-conjugated antibody against the target protein in a compatible buffer overnight at 4°C with rotation. Lysate beads were then washed with the washing buffer (10 mM Tris-HCl, pH 8.0, 1 M NaCl, 1 mM EDTA, 1% NP-40) three times. 2.5 µg purified human protein His-PD-L1 was used for incubation with the above immunocomplex overnight. After washing three times, the lysate beads with IP lysis were boiled for 10 min with 2×SDS loading buffer. Samples were loaded into SDS-PAGE gel for immunoblotting analysis following western blot protocol.

### Immunofluorescence

Double fluorescence staining was performed as we previously described.^22^ Briefly, 4 mm paraffin sections of patient samples were baked for 120 min at 60°C and then deparaffinized. Antigen was retrieved at EDTA antigen retrieval buffer (pH 8.0) and maintain at a sub-boiling temperature for 8 min, standing for 8 min and then followed by another sub-boiling temperature for 7 min. After spontaneous fluorescence quenching, the samples were blocked in 3% BSA, PBS with 0.25% Triton X-100 for 1 hour at room temperature. Primary antibodies targeting (PD-L1, UBQLN4) were incubated overnight at 4°C in the blocking solution and the following day for 30 min at room temperature. After extensive washing in PBS-0.25% Triton X-100, the secondary antibody including anti-rabbit Alexa Fluor 488 or Cy3 dye conjugate (Jackson) was added to the blocking solution and incubated for 2 hours. Then incubate with DAPI solution at room temperature for 10 min, kept in dark place. Images were detected and captured by Fluorescent Microscopy (Nikon, ECLIPSE Ts2R).

### Cell transfection

The 2×10^5^ cells per well were harvested and planted into 6-well plates and culture for 24 hours. when the cells density reaches 80%–90% confluency, UBQLN4-siRNA was transfected into cancer cells using RiboFECT CP Transfection Kit buffer (1×) following product manual protocol. The sequences of human UBQLN4-siRNAs and Control siRNA were listed in online supplemental table 1. After 48 hours transfection, cancer cells were collected and used for total protein and mRNA expression detection.

HEK293T cells with an 80% confluency were transfected for packaging of lentiviral viruses using Turbofect according to the manufacturer’s instruction. After 48 hours and 72 hours transfection, supernatant with viruses was harvested and centrifuged at 2000 rpm for 10 min perspectively. When it was used for infecting cancer cells (30% confluency) in the presence of 8 µg/mL polybrene. After infection 48 hours, cancer cells were subjected to puromycin selection (1 µg/mL) for 3 days for obtaining the stable transfected cells.

### Plasmid construction

pLV-mPuro-C-HA-UBQLN4 constructed by cloning the corresponding cDNAs into pLV-mPuro-C-HA vector was purchased from Sino Biological(HG21666-CYLP, Beijing, China). For CRISPR-mediated knockout A375 cell lines, sgRNAs were subcloned into pLenti CRISPRV2 vector (Addgene) following manufacturer instructions. The sequences of human sgUBQLN4#1 and #2 were listed in online supplemental table 1.

### Data collection and processing

Gene expression profiles (including raw read counts and FPKM) of 33 cancer types were downloaded by R package TCGA biolinks.^23^ Differential expressed genes were identified using limma (|log2(fold change)|>0.58 and BH-adjusted pvalue <0.05).^24^ Other published expression data for patients with ICIs were downloaded from Gene Expression Omnibus.^1 25–33^ The protein expression profile for melanoma patients with ICIs was downloaded from online supplemental materials. For this cohort, the samples treated with anti-PD-1 and taken before treatment were considered, resulting in 66 samples in this cohort.^34^

Gene set variation analysis was performed to calculate the score of immune cell populations.^35^ The gene signature of immune cell populations was obtained from Charoentong *et al*.^36^ Spearman’s correlation test was performed between the expression of interest genes and immune features, considering p<0.05 for statistical significance.

### Statistical analysis

For the tumor growth data analysis, an overall difference at each data collection time point was tested by one-way analysis of variance (ANOVA). No experiment showed obvious bias toward a specific group in starting tumor volume. For comparisons among specific pairs of groups, statistical significance was assessed by the one-way ANOVA followed by Tukey’s multiple comparisons test. The assumption of ANOVA testing was checked to ensure the model assumption is not severely violated. Overall survival analyses were performed using the R package survival and survminer. Patients were divided into two groups by median expression or specified parameters (~45% quantile calculated by maxstat algorithm for protein profile of melanoma cohort with anti-PD-1 therapy). Log-rank tests were performed to compare overall survival between different groups.

## Results

### ABZ enhances CTL activity in association with decrease of tumor PD-L1 expression

**Figure 1:**
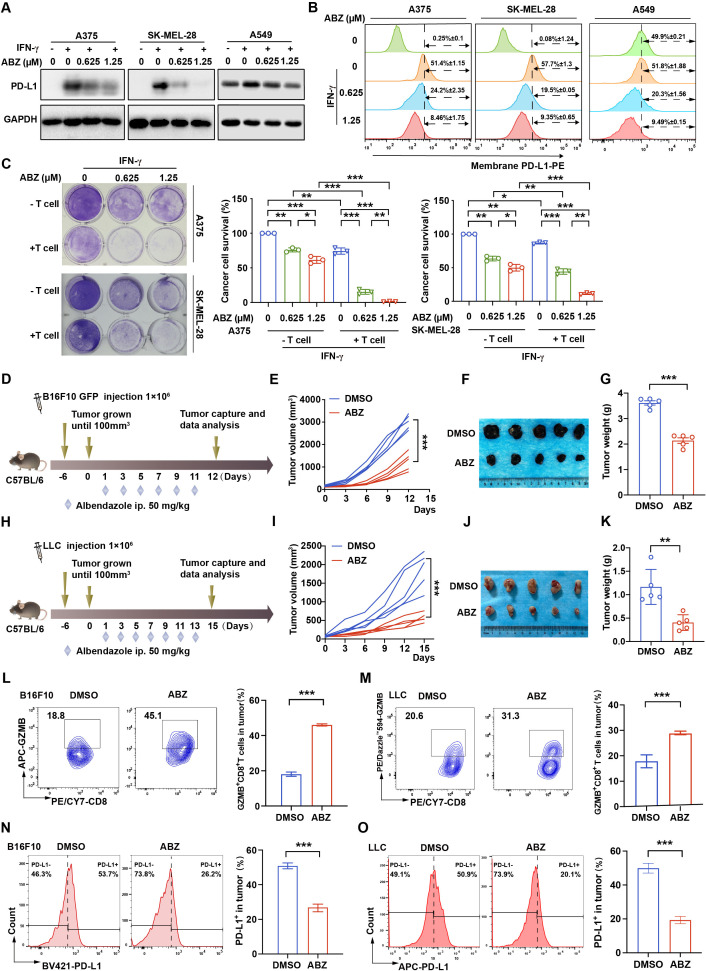
ABZ enhances CTL activity in association with decrease of tumor PD-L1 expression. (A) Representative western blotting of PD-L1 protein level in A375, SK-MEL-28 and A549 cells treated with increasing concentrations of ABZ (0.625–1.25 µM) for 24 hours under IFN-γ exposure. (B) Representative profiles and quantitative analysis of membrane PD-L1 expression by flow-cytometric analysis after increasing concentrations of ABZ (0.625–1.25 µM) treated A375, SK-MEL-28 and A549 cells for 24 hours under IFN-γ exposure. (C) A375 and SK-MEL-28 cells co-cultured with activated T cells for 24 hours with or without ABZ (0.625–1.25 µM) were subjected to crystal violet staining. The tumor cell to T cell ratio, 1:3. The quantitative analysis of A375 and SK-MEL-28 cell survive rate from three independent experiments and showed as means±SD, *p<0.05, **p<0.01, ***p<0.001. (D–G). C57BL/6 mice were implanted with 1×10^6^ B16F10 and received 50 mg/kg of ABZ treatment. (D) A schematic view of the treatment plan. (E) Tumor volume was measured on the indicated different time points. (F, G) Photographs of representative tumors and tumor weight were measured after ABZ treatment on day 12 in the B16F10 tumor burden mouse model. Data represent mean±SD, ***p<0.001. (H-K) C57BL/6 mice were implanted with 1×10^6^ LLC and received 50 mg/kg of ABZ treatment. (H) A schematic view of the treatment plan. (I) Tumor volume was measured on the indicated different time points. (J, K) Photographs of representative tumors and tumor weight were measured after ABZ treatment on day 15 in the LLC tumor burden mouse model. Data represent mean±SD, **p<0.01. (L, M) Representative profiles and quantification of flow cytometry-based detection of the GZMB^+^ CD8^+^ in B16F10 and LLC tumor mass from the different treatment groups (n=5 mice per group). Data represent mean±SD, ***p<0.001. (N, O) Representative profiles and quantification of flow cytometry-based detection of the PD-L1 in B16F10 and LLC tumor mass from the different treatment groups (n=5 mice per group). Data represent mean±SD, ***p<0.001. ABZ, albendazole; CTL, cytotoxic T lymphocyte; IFN-γ, interferon gamma; LLC, Lewis lung cancer.

To find novel therapeutic agents to combine with ICIs for tumor therapy, and because the tumor PD-L1 level is a determinant factor for anti-PD-L1/PD-1 therapy,^22^ we screened a common clinical drug library containing eight imidazoles, which have not been reported in anti-tumor immunity, to investigate their effects on tumor PD-L1 expression. We found that only ABZ significantly reduced the expression of tumor cell membrane PD-L1 levels (online supplemental figure 1A). To further assess whether ABZ regulates tumor PD-L1 levels in vitro, human melanoma cells A375 and SK-MEL-28 and human lung cancer cells A549 were pretreated with IFN-γ to increase PD-L1 levels, human lung cancer cell line H460 and mouse melanoma cells B16F10, which were highly expressed PD-L1 without IFN-γ treatment, followed by treatment with ABZ. Indeed, the protein levels of PD-L1 significantly decreased in an ABZ dose-dependent manner (figure 1A, online supplemental figure 1B, C). Flow cytometry analysis also showed that the IFN-γ-induced increases in membrane PD-L1 levels were decreased in a dose-dependent manner after treatment with ABZ in A375, SK-MEL-28 and A549 cells (figure 1B). Similarly, ABZ treatment significantly decreased the membrane PD-L1 levels in H460 and B16F10 cells without IFN-γ treatment (online supplemental figure 1D). In addition, a T cell killing assay was performed to test the effect of ABZ-associated expression level changes in tumor PD-L1 on CTL activity. As expected, ABZ pretreatment significantly enhanced T cell-mediated cancer cell death (figure 1C, online supplemental figure 1E). However, the expression of PD-1 on CD8^+^ T cells has no significant change after ABZ treatment, indicating that ABZ activates CTL by downregulating the level of tumor PD-L1 (online supplemental figure 1F). To investigate the role of ABZ in anti-tumor immunity, we treated the melanoma B16F10 (figure 1D) and the LLC (figure 1H) tumor-bearing immune-competent mice with ABZ and showed that ABZ treatment significantly reduced mouse tumor growth and weight (figure 1E–G, I–K). Administration of ABZ did not result in a significant change of mouse body weight, the blood routine examination, the morphology and the organizational structure of kidney, liver, and spleen of mice (online supplemental figure 2A-E), suggesting limited toxicity of ABZ treatment. Because immunity-based tumor elimination depends primarily on the activated CD8^+^ T cell, which exerts cytolytic capacity by secreting granzyme B (GZMB), we investigated the infiltrated CD8^+^ T cell abundance and activity in the tumor regions of ABZ-treated mice. Indeed, ABZ treatment significantly increased the CD8^+^ T cell proportion (online supplemental figure 2F, G) and GZMB^+^ CD8^+^ T cell proportion (figure 1L, M) in both melanoma B16F10 and LLC tumor-bearing immune-competent mouse models. The activity of CD8^+^ T cells is mainly controlled by immune checkpoints. Interestingly, we found that ABZ treatment significantly decreased the tumor PD-L1 level (figure 1N, O) but did not affect the levels of PD-1 and CTLA-4 on CD8^+^ T cells (online supplemental figure 2H, I). Together, these data suggested that ABZ increases CTL activity by reducing tumor PD-L1 levels.

### ABZ induces ubiquitin-mediated PD-L1 degradation

**Figure 2:**
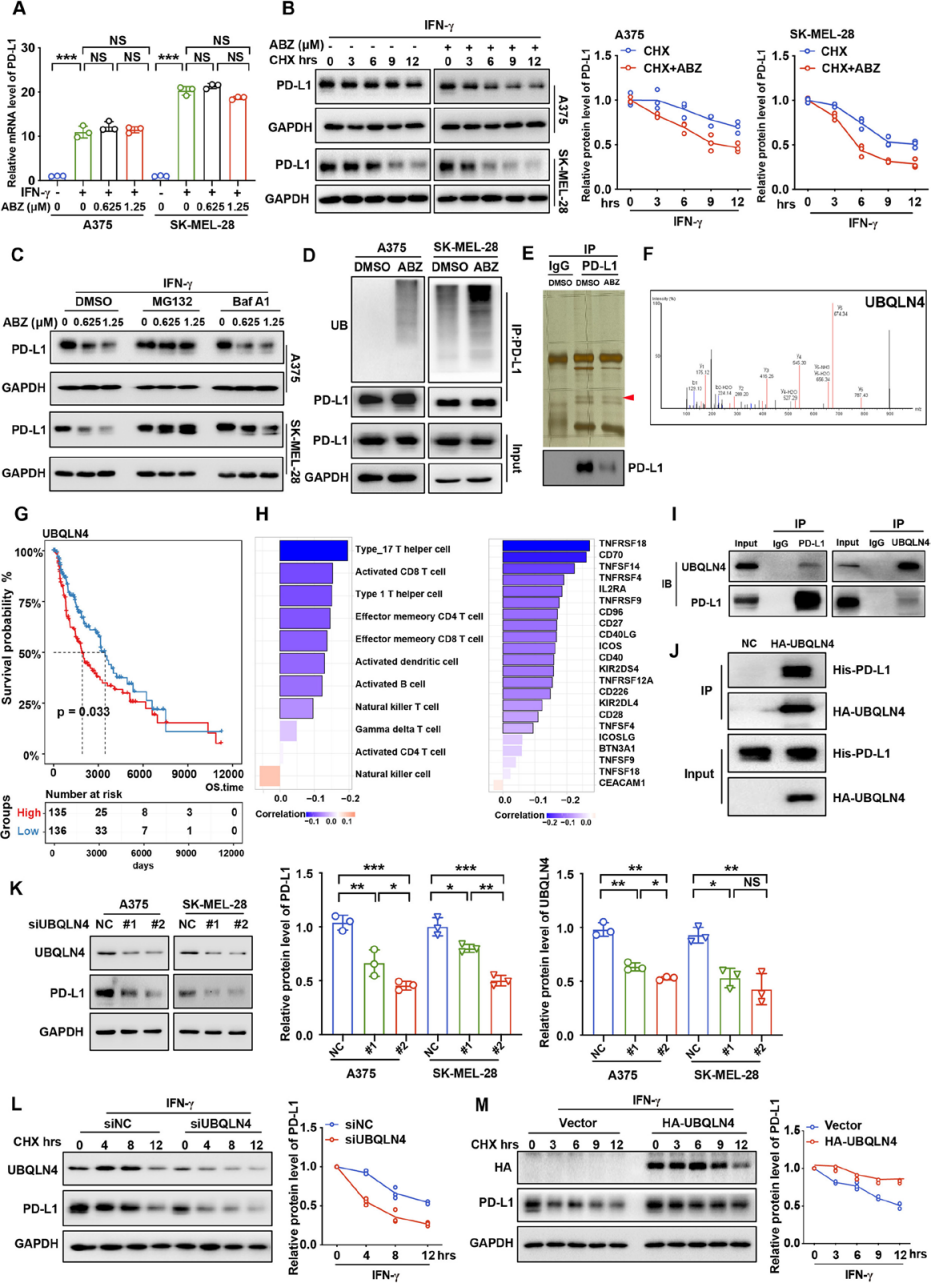
ABZ decreases UBQLN4 and induces PD-L1 ubiquitin-mediated degradation. (A) Bar graph presentation of PD-L1 mRNA levels as determined by qRT-PCR of increasing concentrations of ABZ (0.625–1.25 µM) treatment of A375 and SK-MEL-28 cells for 24 hours under IFN-γ exposure, each bar represents the mean±SD of three independent experiments, ^NS^P >0.05, ***p<0.001. (B) A375 and SK-MEL-28 cells were treated with ABZ (1.25 µM) for 24 hours under IFN-γ exposure then treated with CHX (200 ng/mL), and collected at the indicated times for western blotting. Line graph presentation of quantitative analysis of PD-L1 protein expression in A375 and SK-MEL-28 cells. (C) A375 and SK-MEL-28 cells were pretreated with increasing concentrations of ABZ (0.625–1.25 µM), then treated with Baf A1 (100 nM) and MG132 (10 µM) for 6 hour and the PD-L1 protein level were detected by western blotting. (D) A375 and SK-MEL-28 cells were treated with ABZ (1.25 µM) for 24 hours under IFN-γ exposure then treated with MG132 (10 µM) for 6 hours before harvest. PD-L1 was immunoprecipitated with an anti-PD-L1 antibody, and the immunoprecipitates were probed with an anti-ubiquitin (UB) antibody. (E) Immunoprecipitates from A375 cells treated with ABZ (1.25 µM) or DMSO were separated by SDS-PAGE and the immunoprecipitates were probed with anti-PD-L1 antibody and stained with colloidal silver to visualize proteins, respectively. The proteins pointed by the arrow were analyzed by LC/MS-MS. (F) Peptide enrichment fingerprints of UBQLB4 from LC/MS-MS QSTAR analysis. (G) Kaplan-Meier estimates for overall survival; patients from TCGA SKCM cohort were stratified into two groups based on the median expression level of UBQLN4. Significance was determined by the log-rank test. (H) Significantly correlation between UBQLN4 expression level and infiltration of activated immune cell (left panel) or expression level of activated checkpoints (right panel) in TCGA SKCM cohort, respectively. Spearman’s correlation test was performed, black border, *p<0.05. (I) A375 cell lysates were subjected to immunoprecipitation with control IgG, anti-PD-L1 or anti-UBQLN4 antibodies. The immunoprecipitates were then probed with anti-UBQLN4 or anti-PD-L1 antibody, respectively. (J) Representative western blotting analysis of pull-down of purified HA-UBQLN4 with purified His-PD-L1. (K) Representative western blotting and quantitative analysis of UBQLN4 and PD-L1 proteins expression when knockdown UBQLN4 in A375 and SK-MEL-28 cells under IFN-γ exposure, each bar represents the mean±SDof three independent experiments, ^NS^P >0.05, *p<0.05, **p<0.01, ***p<0.001. (L) A375 cells transfected with control or UBQLN4 siRNA were treated with CHX (200 ng/mL) and collected at the indicated times for western blotting. Line graph presentation of quantitative analysis of PD-L1 protein level. (M) A375 cells transfected with a vector expressing HA-UBQLN4 and an empty vector were treated with CHX (200 ng/mL) and collected at the indicated times for western blotting. Line graph presentation of quantitative analysis of PD-L1 protein level, GAPDH was used as the loading control. ABZ, albendazole; IFN-γ, Interferon gamma; CHX, cycloheximide; LC/MS-MS, liquid chromatography tandem mass spectrometry; GAPDH, glyceraldehyde-3-phosphate dehydrogenase.

Next, we aim to explore the molecular mechanism of ABZ-mediated regulation of PD-L1. Our results showed that the mRNA levels of PD-L1 did not significantly change after ABZ treatment (figure 2A), indicating that ABZ may regulate PD-L1 expression via protein-level modifications rather than at the transcriptional level. Furthermore, melanoma cells were treated with cycloheximide (CHX) to inhibit protein biosynthesis, and protein extracts obtained at the indicated time points were analyzed. We found that ABZ treatment significantly decreased the half-life of the PD-L1 protein in A375 and SK-MEL-28 cells (figure 2B), suggesting that ABZ regulates the stability of PD-L1 protein. Interestingly, the reduction in PD-L1 protein levels was blocked by the proteasome inhibitor MG132 but not by the lysosome inhibitor bafilomycin A1 (Baf A1, figure 2C, online supplemental figure 3A), suggesting that ABZ induces degradation of PD-L1 protein via the ubiquitin-proteasome system (UPS) rather than via the autophagy/lysosome pathway. To further confirm that ABZ induces degradation of the PD-L1 protein via the UPS pathway, we measured the levels of polyubiquitylated PD-L1 in melanoma cells. As shown in figure 2D, ABZ treatment led to a significant increase in PD-L1 polyubiquitylation. Taken together, these data indicated that ABZ treatment induces PD-L1 ubiquitin-mediated degradation.

### UBQLN4 interacts with PD-L1 and stabilizes the PD-L1 protein

To further investigate the molecular mechanism underlying ABZ-induced ubiquitin-mediated degradation of PD-L1, we performed immunoprecipitation coupled to mass spectrometry in A375 cells with ABZ treatment (figure 2E). UBQLN4 was identified as the top candidate in terms of enrichment of peptides among all the candidate ubiquitylation-related proteins (figure 2F, online supplemental table 3). UBQLN4 has been reported to function as a UBL-UBA protein (proteins that contain both a ubiquitin-like region and ubiquitin-associated domain) that stabilizes its target substrate, wild-type ataxin-1-(30Q).^37^ More importantly, UBQLN4 was significantly correlated with worse survival (figure 2G) and negatively correlated with activated CTLs and immune checkpoints in melanoma (figure 2H, online supplemental figure 3B). To confirm the interaction between UBQLN4 and PD-L1, co-IP was performed and revealed the endogenous interaction of UBQLN4 with PD-L1 in A375 cells (figure 2I). Furthermore, a pull-down assay showed that purified HA-UBQLN4 protein was directly bound to His-PD-L1 protein (figure 2J). Given the identified interaction of UBQLN4 with PD-L1, we next investigated whether UBQLN4 affects the protein level of PD-L1. Interestingly, knockdown of UBQLN4 using two siRNA sequences significantly decreased PD-L1 protein levels in A375 and SK-MEL-28 cells (figure 2K). More importantly, we found that the knockdown of UBQLN4 significantly decreased the half-life of the PD-L1 protein (figure 2L). In contrast, overexpression of UBQLN4 significantly increased the half-life of the PD-L1 protein (figure 2M). Together, these data suggest that UBQLN4 interacts with PD-L1 and stabilizes the PD-L1 protein.

**Figure 3:**
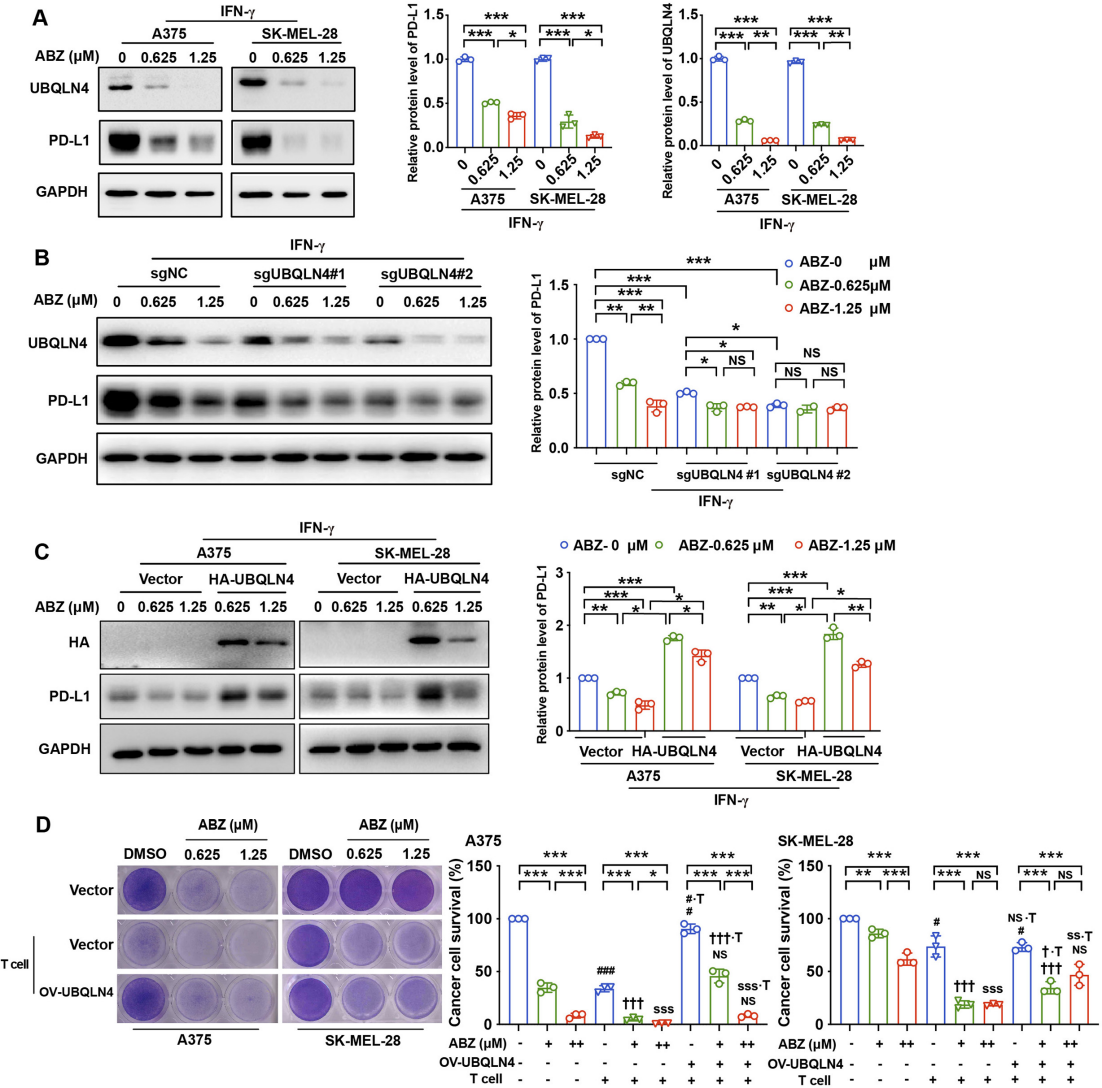
ABZ reduces PD-L1 and enhances CTL activity by downregulating UBQLN4. (A) Representative western blotting and quantitative analysis of UBQLN4 and PD-L1 protein levels in A375 and SK-MEL-28 cells treated with increasing concentrations of ABZ (0.625–1.25 µM) for 24 hours under IFN-γ exposure, each bar represents the mean±SD of three independent experiments, *p<0.05, **p<0.01, ***p<0.001. (B) Representative western blotting and quantitative analysis of PD-L1 protein levels in A375, which were stable transfected with sgRNAs (sgUBQLN4 #1, sgUBQLN4 #2 and control (NC)) and treated with increasing concentrations of ABZ (0.625-1.25 µM), each bar represent the mean±SD of three independent experiments, ^NS^P >0.05, *p<0.05, **p<0.01, ***p<0.001. (C) Representative western blotting and quantitative analysis of PD-L1 protein levels in A375 and SK-MEL-28 cells, which were transfected with HA-UBQLN4 and empty vector and treated with increasing concentrations of ABZ (0.625–1.25 µM), each bar represents the mean±SD of three independent experiments, *p<0.05, **p<0.01, ***p<0.001. (D) A375 and SK-MEL-28 cells were transfected with HA-UBQLN4 and empty vector and cocultured with activated T cells for 24 hours with or without ABZ (0.625-1.25 µM) were subjected to crystal violet staining. The tumor cell to T cell ratio, 1:3. The quantitative analysis of A375 and SK-MEL-28 cells survive rate from three independent experiments and showed as means±SD, ^NS^P >0.05, *p<0.05, **p<0.01, ***p<0.001. ^#^p<0.05, ^###^p<0.001, vs the DMSO group. ^†††^p<0.001 vs the ABZ (0.625 µM) group. ^SSS^p <0.001 vs the ABZ (1.25 µM) group. ^NS∙T^p >0.05, ^#∙T^p <0.05, vs the DMSO +T group. ^†∙T^p <0.05, ^†††∙T^p <0.001 vs the ABZ (0.625 µM+T) group. ^SS∙T^p <0.001, ^SSS∙T^ p<0.001 vs the ABZ (1.25 µM+T) group. ABZ, albendazole; IFN-γ, interferon gamma.

To validate that UBQLN4 is a functional target of ABZ and is responsible for reducing PD-L1 expression and enhancing CTL activity, we first treated A375, SK-MEL-28 and H460 cells with ABZ and found that the expression of UBQLN4 was significantly decreased, and the PD-L1 levels were also decreased (figure 3A, online supplemental figure 3C). Interestingly, knockout of UBQLN4 using clustered regularly interspaced short palindromic repeats (CRISPR)-CRISPR-associated protein 9 (Cas9) technology with the single guide RNAs (sgRNAs) sgUBQLN4 #1 and sgUBQLN4 #2 abolished the ABZ treatment-induced reduction in PD-L1 protein levels (figure 3B). However, overexpression of UBQLN4 strongly reversed the ABZ-mediated decrease in PD-L1 (figure 3C). In addition, a T cell killing assay also showed that ABZ pretreatment-induced enhancement of T cell-mediated cancer cell death was rescued by overexpression of UBQLN4 (figure 3D). These results suggested that UBQLN4 is a functional target of ABZ and is required for ABZ to reduce PD-L1 and enhance CTL activity.

### Genetic deletion of CD73 enhanced ABZ-mediated immunotherapy response in vivo

**Figure 4:**
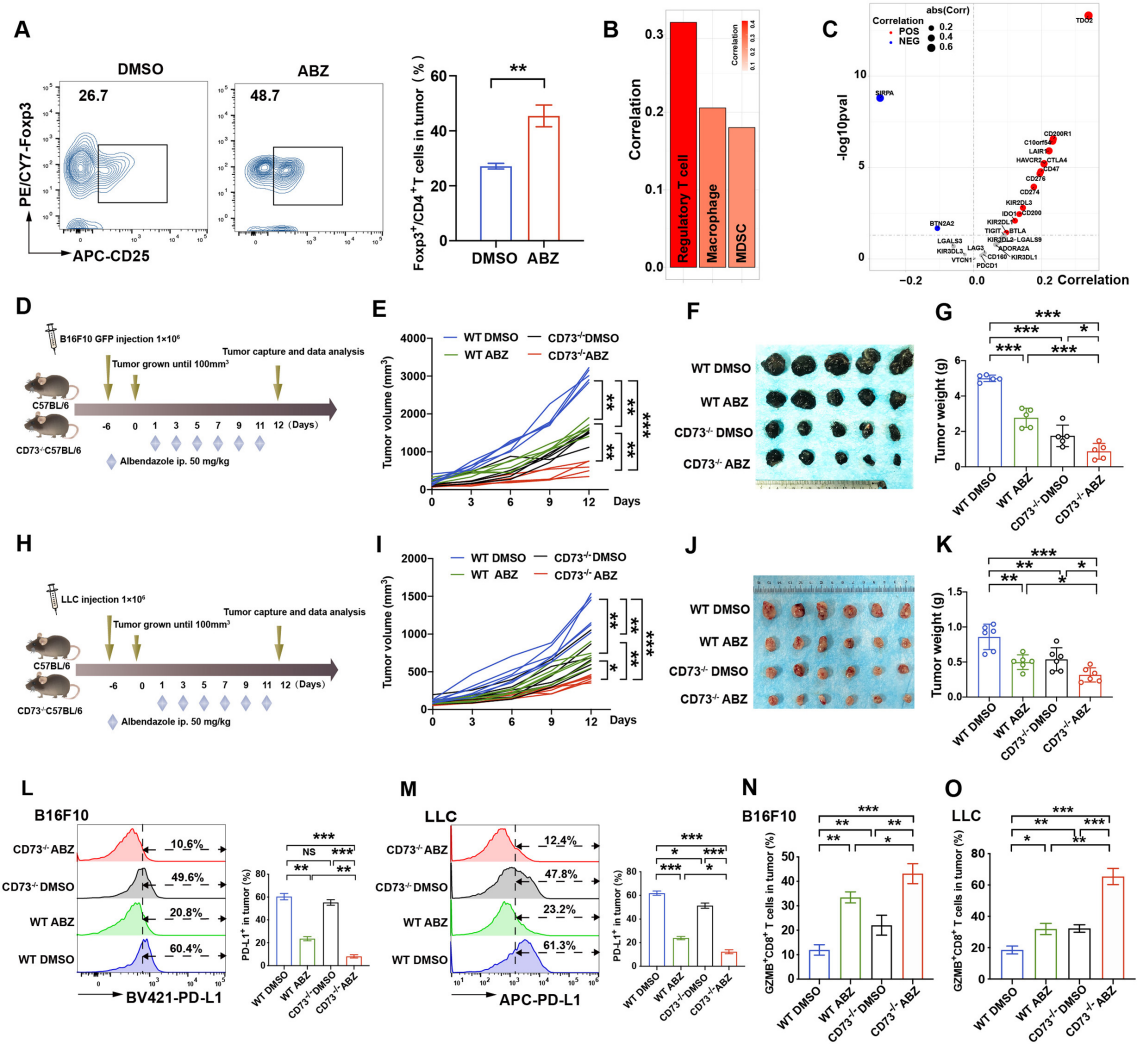
Genetic deletion of CD73 enhanced ABZ-mediated immunotherapy response in vivo. (A) Representative profiles and quantification of flow cytometry-based detection of the Foxp3^+^CD4^+^ TILs in B16F10 tumor mass from the different treatment groups (n=5 mice per group, **p<0.01). (B, C) Significantly correlation for suppressive immune cell types or inhibitory immune checkpoints and CD73 expression level in TCGA SKCM cohort. Spearman’s correlation test was performed. Bars with black border, *p<0.05. Points in the scatter plot represent positive (RS >0 and pvalue <0.05, in red), negative (RS <0 and pvalue <0.05, in blue), or non-significant correlation (in gray). (D–G) CD73 WT and KO mice (n=5 mice per group) were implanted with 1×10^6^ WT B16F10 and received 50 mg/kg of ABZ treatment as the chart indicated. (D) a schematic view of the treatment plan. (E) Tumor volume was measured on the indicated different time points (n=5 mice per group). Data represent mean±SD, **p<0.01, ***p<0.001. (F) Photographs of representative tumors harvested from CD73 WT and KO mice after ABZ treatment at day 12. (G) Tumor weight was measured on day 12. Data represent mean±SD, *p<0.05, ***p<0.001. (H–K) CD73 WT and KO mice (n=6 mice per group) were implanted with 1×10^6^ WT LLC and received 50 mg/kg of ABZ treatment as the chart indicated. (H) A schematic view of the treatment plan. (I)Tumor volume was measured on the indicated different time points (n=6 mice per group). Data represent mean±SD, *p<0.05, **p<0.01, ***p<0.001. (J) Photographs of representative tumors harvested from CD73 WT and KO mice after ABZ treatment at day 12. (K) Tumor weight was measured on day 12. Data represent mean±SD, *p<0.05, **p<0.01, ***p<0.001. (L, M) representative profiles and quantification of flow cytometry-based detection of the PD-L1 from B16F10 (L) and LLC (M) tumor mass (n=5 mice per group). Data represent mean±SD, ^NS^P >0.05, **p<0.01, ***p<0.001. (N, O) quantification of flow cytometry-based detection of the GZMB^+^ CD8^+^ in CD3^+^ TILs from B16F10 (N) and LLC (O) tumor mass (n=5 mice per group). Data represent mean±SD, *p<0.05, **p<0.01, ***p<0.001. ABZ, albendazole;TILs, tumor infiltrates lymphocytes; LLC, Lewis lung cancer.

ABZ increased CD8^+^ T cell cytotoxicity while elevating Treg cell abundance as an additional effect in vivo (figure 4A). Bioinformatics analysis showed that CD73 expression was significantly positively correlated with immunosuppressive cells (Treg cells, macrophages and MDSCs) and inhibitory immune checkpoints (figure 4B, C, online supplemental figure 4A, B). Moreover, blocking CD73 has been shown to enhance antitumor responses in a number of tumor models.^38^ Therefore, we further investigated whether deficiency of CD73, a checkpoint molecule expressed on Treg cells,^15^ could synergize with the tumor elimination effect of ABZ in immune-competent mice. We examined the tumor elimination effects of ABZ in immune-competent CD73 wild-type (WT) and knockout (KO) mice bearing B16F10 (figure 4D) and LLC tumors (figure 4H). We observed that the growth of B16F10 and LLC subcutaneous tumors was significantly inhibited in ABZ-treated KO mice in comparison with that in ABZ- or DMSO-treated WT mice and CD73 KO mice (figure 4E, I). Moreover, ABZ-treated CD73 KO mice displayed a significantly greater reduction in tumor mass and weight than ABZ-treated or DMSO-treated WT mice and CD73 KO mice (figure 4F, G, J, K) without significant changes in body weight, the blood routine examination, the morphology of lung, liver, and spleen of mice in both melanoma B16F10 and LLC tumor-bearing immune-competent mouse models (online supplemental figure 4C–H). As expected, ABZ-treated CD73 KO mice showed further decreased PD-L1 expression levels in the B16F10 and LLC subcutaneous tumor regions (figure 4L, M) and increased proportions of infiltrated CD8^+^ and GZMB^+^ CD8^+^ T cells in the tumor region (figure 4N, O, online supplemental figure 5A-D). More importantly, ABZ-treated CD73 KO mice exhibited a markedly decreased Treg population compared with ABZ- or DMSO-treated WT mice and CD73 KO mice (online supplemental figure 5E).

### Synergistic effect of CD73 specific inhibitor and ABZ therapy in the melanoma mouse model

**Figure 5:**
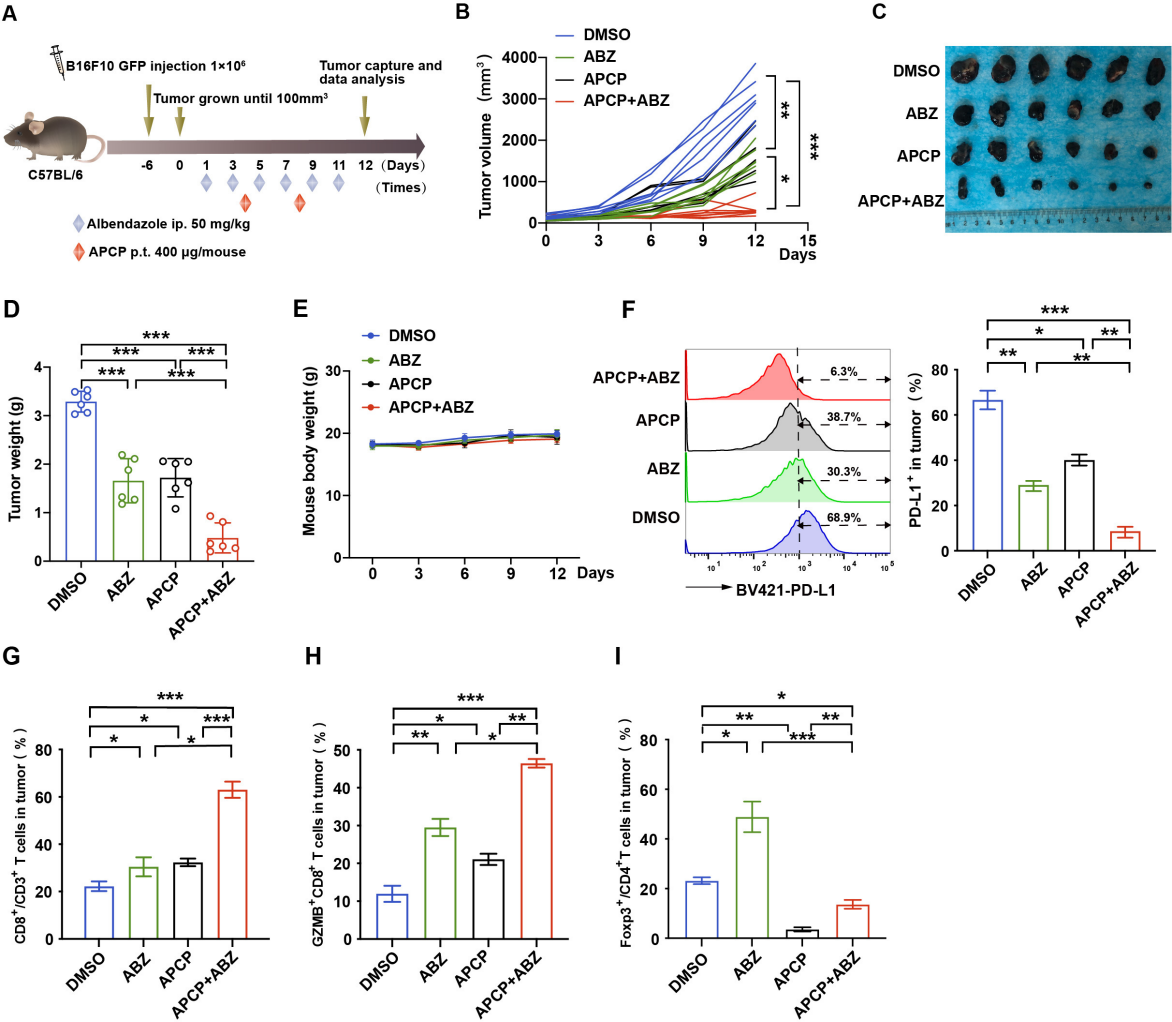
Synergistic effect of CD73 specific inhibitor and ABZ therapy in the melanoma mouse model. (A) Mouse melanoma cell line B16F10 cells were injected into mice (n=6 mice per group) on day −6, ABZ (50 mg/kg) and APCP (400 µg/mouse) were administered alone or together as the chart indicated. (B) Tumor volume was measured on the indicated different time points (n=6 mice per group). Data represent mean±SD, *p<0.05, **p<0.01, ***p<0.001. (C) Photographs of representative tumors after ABZ and APCP treatment on day 12 in the B16F10 tumor burden mouse model. (D) Tumor weight was measured on day 12. Data represent mean±SD, ***p<0.001. (E) Mice weight was measured on the indicated different time points. (F) Representative profiles and quantification of flow cytometry-based detection of the PD-L1 from B16F10 tumor mass (n=6 mice per group). Data represent mean±SD, *p<0.05, **p<0.01, ***p<0.001. (G) Representative profiles and quantification of flow cytometry-based detection of the CD8^+^ in CD3^+^ TILs in B16F10 tumor mass from the different treatment groups (n=6 mice per group). Data represent mean±SD, *p<0.05, ***p<0.001. (H) Representative profiles and quantification of flow cytometry-based detection of the GZMB^+^ CD8^+^ in CD3^+^ TILs from the B16F10 tumor mass (n=6 mice per group). Data represent mean±SD, *p<0.05, **p<0.01, ***p<0.001. (I) Representative profiles and quantification of flow cytometry-based detection of the Foxp3^+^ in CD4^+^ TILs in B16F10 tumor mass from the different treatment groups (n=6 mice per group). Data represent mean±SD, *p<0.05, **p<0.01, ***p<0.001. ABZ, albendazole; APCP, adenosine 5'-(α,β-methylene) diphosphate; TILs, tumor infiltrates lymphocytes.

We further identified whether combining ABZ with a CD73-specific inhibitor (APCP) can enhance tumor therapeutic efficacy. To test this hypothesis, B16F10 tumor-bearing mice were treated with ABZ, APCP, ABZ plus APCP, or the control (figure 5A). Notably, combining ABZ and APCP reduced tumor growth and tumor weight much more significantly than either treatment alone (figure 5B–D) without causing significant changes in body weight (figure 5E). Similar to the combination of ABZ and CD73 deficiency, cotreatment with ABZ and APCP synergistically decreased PD-L1 expression levels in the tumor regions (figure 5F). Notably, the combination of ABZ and APCP also markedly increased the population of infiltrated CD8^+^ and GZMB^+^ CD8^+^ T cells in the tumor region (figure 5G, H). Moreover, cotreatment with ABZ and APCP synergistically decreased the Treg cell populations compared with ABZ or APCP treatment alone (figure 5I). These results indicate that ABZ reduces tumor PD-L1 levels and enhances the efficacy of CD73 blockade immunotherapy.

### UBQLN4/PD-L1 expression levels were correlated with the efficacy of PD-1 mAb therapy in melanoma patients

**Figure 6:**
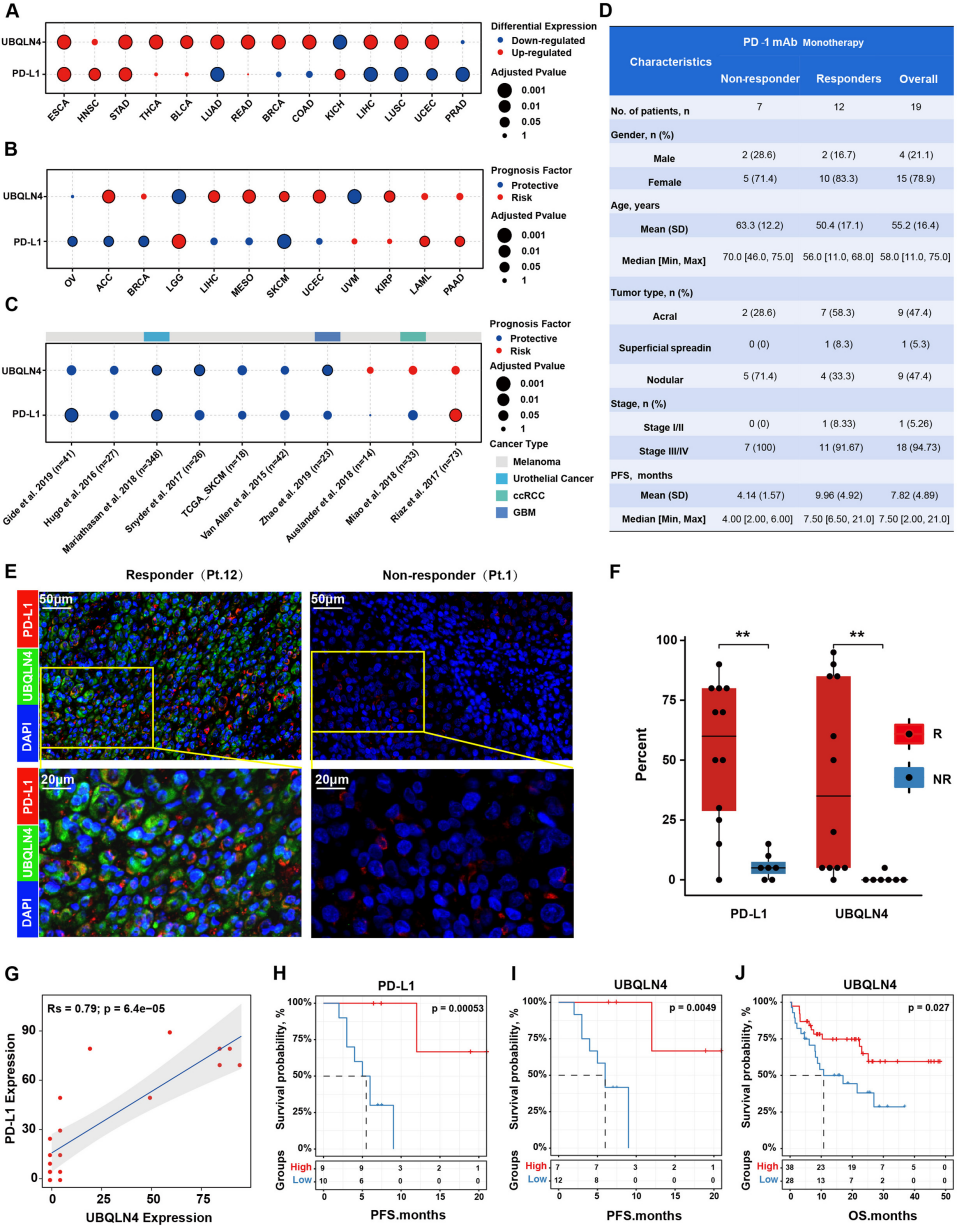
UBQLN4/PD-L1 expression levels were correlated with the efficacy of PD-1 mAb therapy in melanoma patients. (A) Differential expression of UBQLN4 and PD-L1 across 16 cancer types compared with paired normal samples (fold change >1.5; adjusted p<0.05). Cancer types with UBQLN4 or PD-L1 significantly differentially expressed were shown. (B) Survival analysis demonstrated the effect of UBQLN4 and PD-L1 on patient overall survival in traditional therapy strategies. (C) Survival analysis demonstrated the effect of UBQLN4 and PD-L1 on patient overall survival in anti-PD-1 immunotherapy. (D) Clinical characteristics of anti-PD-1 monotherapy cohorts. Patients were stratified into response groups based on RECIST (Response Evaluation Criteria in Solid Tumors) 1.1 criteria, patients with a complete response (CR), partial response (PR), or stable disease (SD) with progression-free survival (PFS) longer than 6 months were classified as responders, while patients with SD with PFS shorter than 6 months and PD were categorized as non-responders. TNM stage based on the eighth Edition Melanoma Stage Classification. (E) Representative fluorescent images of UBQLN4 and PD-L1 in two patients with a different response. (F) Significantly differential expression of UBQLN4 and PD-L1 between non-responder and responder. Significance was determined by the Wilcoxon rank-sum test, **p<0.01. (G) Scatterplot showed a significant correlation between UBQLN4 and PD-L1 expression. (H, I) Kaplan-Meier estimates for PFS of patients derived from Xiangya hospital patients (n=19) were stratified into two groups based on the median expression level of PD-L1 (H) and UBQLN4 (I), respectively. Significance was determined by the log-rank test. (J) Kaplan-Meier estimates for overall survival of patients derived from Harel *et al*,^39^ (n=66).

To investigate the clinical application potential of strategies targeting UBQLN4/PD-L1, we compared the expression levels of UBQLN4/PD-L1 between paired tumor and normal tissue samples across 16 cancer types from The Cancer Genome Atlas (TCGA). We observed that UBQLN4 and PD-L1 were upregulated in most cancer types, including esophageal carcinoma, head and neck squamous cell carcinoma, adenocarcinoma of the stomach and lung adenocarcinoma (figure 6A). We further performed survival analyses and demonstrated that high UBQLN4/PD-L1 scores were associated with worse survival in most cancer types from TCGA, the patients of whom were mainly treated with chemotherapy or targeted therapy (figure 6B). In contrast, we found that the expression levels of UBQLN4/PD-L1 were associated with better survival in most cancer types from the public ICB cohort (figure 6C). One reason that only a small subset of patients respond to PD-1/PD-L1 blockade is that PD-1-associated immune resistance depends on the expression of PD-L1 in the tumor. Therefore, we analyzed the levels of UBQLN4/PD-L1 in biopsy samples from two immunotherapy cohorts. We collected 19 melanoma patients treated with anti-PD-1 monotherapy in Xiangya Hospital (figure 6D, online supplemental table 2). As expected, samples from responders had a relatively higher UBQLN4/PD-L1 signal than the samples from non-responders (figure 6E, F). Meanwhile, a strong positive correlation between the UBQLN4/PD-L1 expression was observed in patient samples (figure 6G). Moreover, we observed that patients with high UBQLN4 and PD-L1 expression in the tumor region had better progression-free survival (PFS; median PFS 10 months vs 2 months, log-rank test, p<0.001) after anti-PD-1 treatment (figure 6H, I). In addition, we further separate patients into three groups based on UBQLN4 and PD-L1 expression, and revealed that patients with status of ‘PD-L1-low and UBQLN4-low’ showed the worst survival (online supplemental figure 6). We also collected published protein expression profiles for 66 melanoma patients treated with anti-PD-1 antibodies.^39^ Similarly, we found that a high protein level of UBQLN4 was correlated with a better OS in Harel *et al*. Dataset (overall Survival, log-rank test, p=0.027, figure 6J). Taken together, these data demonstrated that the expression of UBQLN4/PD-L1 is a promising cancer biomarker and potential predictor for assessing the efficacy of anti-PD-1 therapy in the clinic.

## Discussion

ICIs have shown substantial clinical benefits for several malignancies, such as metastatic melanoma and NSCLC. However, the overall response rate to ICIs has been relatively low.^19^ Previous preclinical studies and clinical trials have also shown that traditional chemotherapeutic drugs combined with ICIs, such as metformin plus CTLA-4 blockade,^40^ sunitinib plus CTLA-4 blockade,^22^ and pembrolizumab plus carboplatin plus pemetrexed (phase II of KEYNOTE-021),^41^ are more effective in reversing T cell exhaustion and restoring antitumor immunity than single-agent treatment. In particular, drug repurposing and/or repositioning is a method for developing new treatments for cancers.^42^ In this study, we revealed a previously unappreciated role of the antiparasitic agent ABZ: it can induce an antitumor immune response by increasing CD8^+^ T cell cytotoxicity. Preclinically, we showed that ABZ had a synergistic effect with CD73 blockade in the treatment of immune-competent mice, providing a novel combination strategy for cancer immunotherapy.

PD-L1 is a critical immune checkpoint protein that binds to the PD-1 receptor on T cells and activates coinhibitory signaling to suppress the function of CTLs, allowing cancer cells to escape immunosuppression.^43^ It is not surprizing that PD-L1 is expressed at relatively much higher levels in many types of cancer cells and is often correlated with poor patient prognosis.^44 45^ Recent studies have reported that the tumor PD-L1 expression level is a determinant and a common biomarker for the assessment of the clinical response to anti-PD-L1/PD-1 therapy.^7^ However, how and when PD-L1 is upregulated during the pathogenesis of cancer remains less well understood. Therefore, it is critical to understand the molecular mechanism of tumor PD-L1 regulation, which is important for the improvement of anti-PD-L1/PD-1 therapy and its subsequent clinical effect. Previous studies have reported that the tumor PD-L1 level can be regulated at the transcriptional level (eg, via EGFR, the MAPK pathway, Janus kinase-signal transducer and activator of transcription pathway, nuclear factor kappa-B (NF-κB) pathway, hypoxia-inducible factor-1α, MYC, Anaplastic lymphoma kinase, Met, BRD4),^46^ and via post-translational modification (eg, via CDK4, B3GNT3, GSK3β, CSN5, CMTM4, and CMTM6).^47^ In this study, we revealed that UBQLN4 interacts with PD-L1 and stabilizes the PD-L1 protein. ABZ induced ubiquitination and degradation of PD-L1 by reducing the expression of UBQLN4. We not only uncovered the molecular mechanisms by which ABZ promotes antitumor immunity but also, more importantly, identified UBQLN4 as a novel posttranslational regulatory molecule of PD-L1.

UBQLN4 belongs to the UBL-UBA family of proteins, which are adaptor proteins that deliver ubiquitinated proteins through the UBA domain to s5a located in the proteasome through simultaneous interaction with the UBL domain and play main roles in protein degradation.^48^ For instance, UBQLN4 interacts with endoplasmic reticulum-localized connexin 43 (Cx43) and stimulates its proteasomal degradation.^49^ However, recent findings have contradictorily reported that UBL-UBA proteins also exert stabilizing effects on their substrates.^50 51^ Recently, some reports have shown that UBQLN4 is overexpressed in neuroblastoma and hepatocellular carcinoma (HCC) tumors and that high UBQLN4 expression is associated with poor overall survival and disease-free survival rates.^52 53^ Herein, we demonstrated that UBQLN4, CD73, and PD-L1 were significantly highly expressed in most cancers and that their high expression correlated with lower survival of cancer patients. These data provide potential new diagnostic and therapeutic targets in cancers. On the other hand, we observed higher UBQLN4 and PD-L1 levels in the tumor region of melanoma patients who responded to anti-PD-1 therapy than in the tumor region of nonresponders, indicating that UBQLN4 and PD-L1 might be potential biomarkers for assessing and predicting the efficacy of anti-PD-1 therapy in the clinic.

In summary, our data revealed a previously uncharacterized function of ABZ in promoting antitumor immunity by disrupting the UBQLN4-mediated stabilization of the PD-L1 protein. Our findings also suggest that ABZ can be exploited to overcome tumor immune escape in combination with current ICIs, such as CD73 inhibitors.

10.1136/jitc-2021-003819.supp1 Supplementary data

## Data Availability

Data are available in a public, open access repository.
