# Supplementary material for: Albendazole induces immunotherapy response by facilitating ubiquitin-mediated PD-L1 degradation
Source: J Immunother Cancer. 2022 May 16;10(5):e003819. doi: 10.1136/jitc-2021-003819 (PMC9115032; doi:10.1136/jitc-2021-003819)
Supplement: Supplementary data [file jitc-2021-003819supp002.pdf]

Supplementary Figure 1

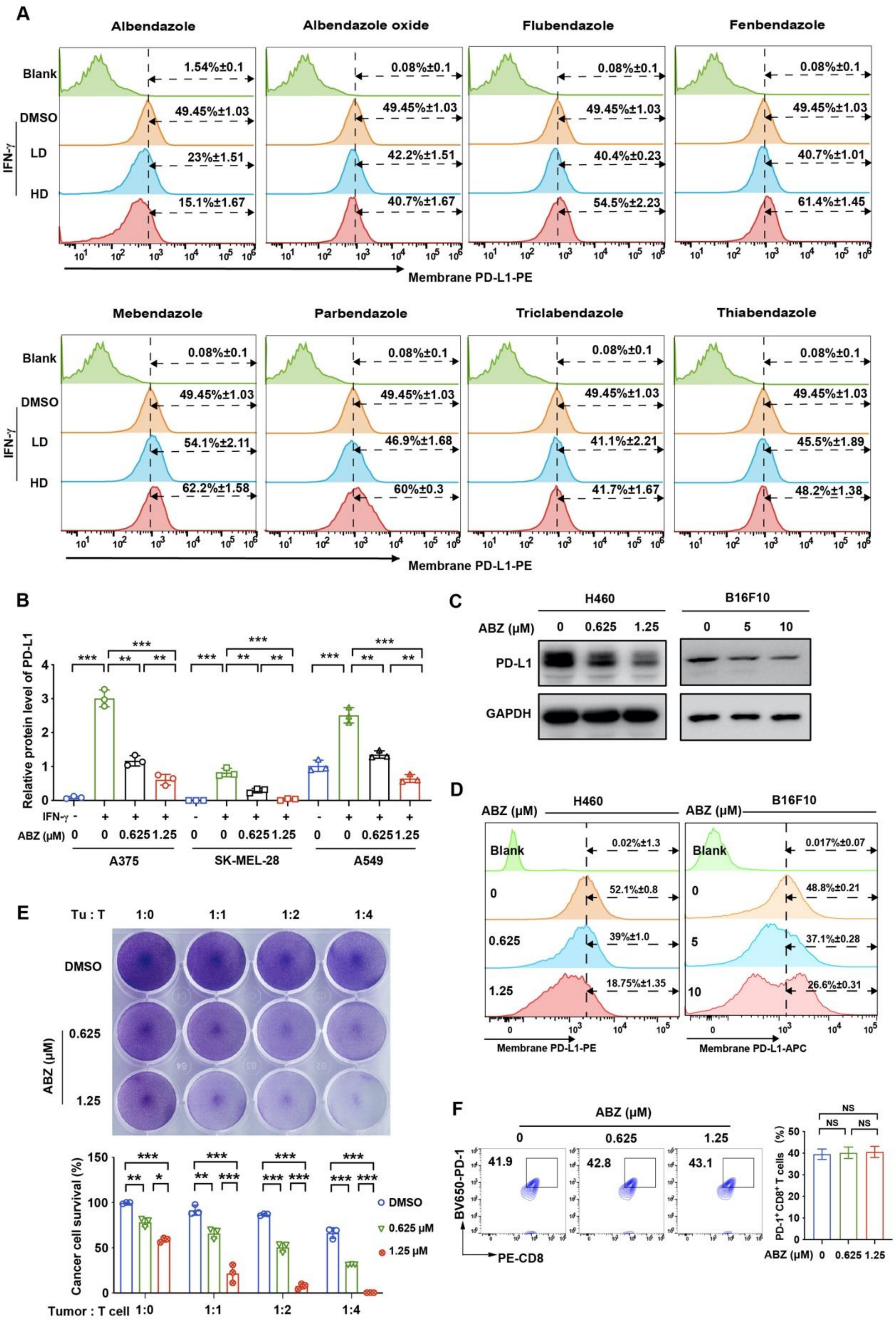

A. Representative profiles and quantitative analysis of flow cytometry analysis of membrane PD-L1 expression in A375 cells treated with low dose (LD) and high dose (HD) of 8 kinds of imidazoles for 24 h under IFN-γ exposure. B. Quantitative analysis of PD-L1 protein level in A375, SK-MEL-28 and A549 treated with increasing concentrations of ABZ (0.625 to 1.25 μM) for 24 h under IFN-γ exposure , each bar represents the mean±S.D of three independent experiments, *p<0.05, **p<0.01, ***p<0.001. C. Representative western blotting of PD-L1 protein level in H460 and B16F10 cells treated with increasing concentrations of ABZ for 24 h. D. Representative profiles and quantitative analysis of membrane PD-L1 expression by flow-cytometric analysis after increasing concentrations of ABZ treated H460 and B16F10 cells for 24 h. E. co-cultured with activated T cells for 24 h with or without ABZ (0.625 to 1.25 μM) were subjected to crystal violet staining. The tumor cell to T cell ratio, 1:0, 1:1, 1:2, 1:4. The quantitative analysis of H460 cell survive rate from three independent experiments and showed as means±SD, *p<0.05, **p<0.01, ***p<0.001. F. Representative profiles and quantification of flow cytometry-based detection of the PD-1^+ in $CD8^+$ TILs after increasing concentrations of ABZ treated T cells for 24 h. Data represent mean±SD, $^{NS}p>0.05$.

Supplementary Figure 2

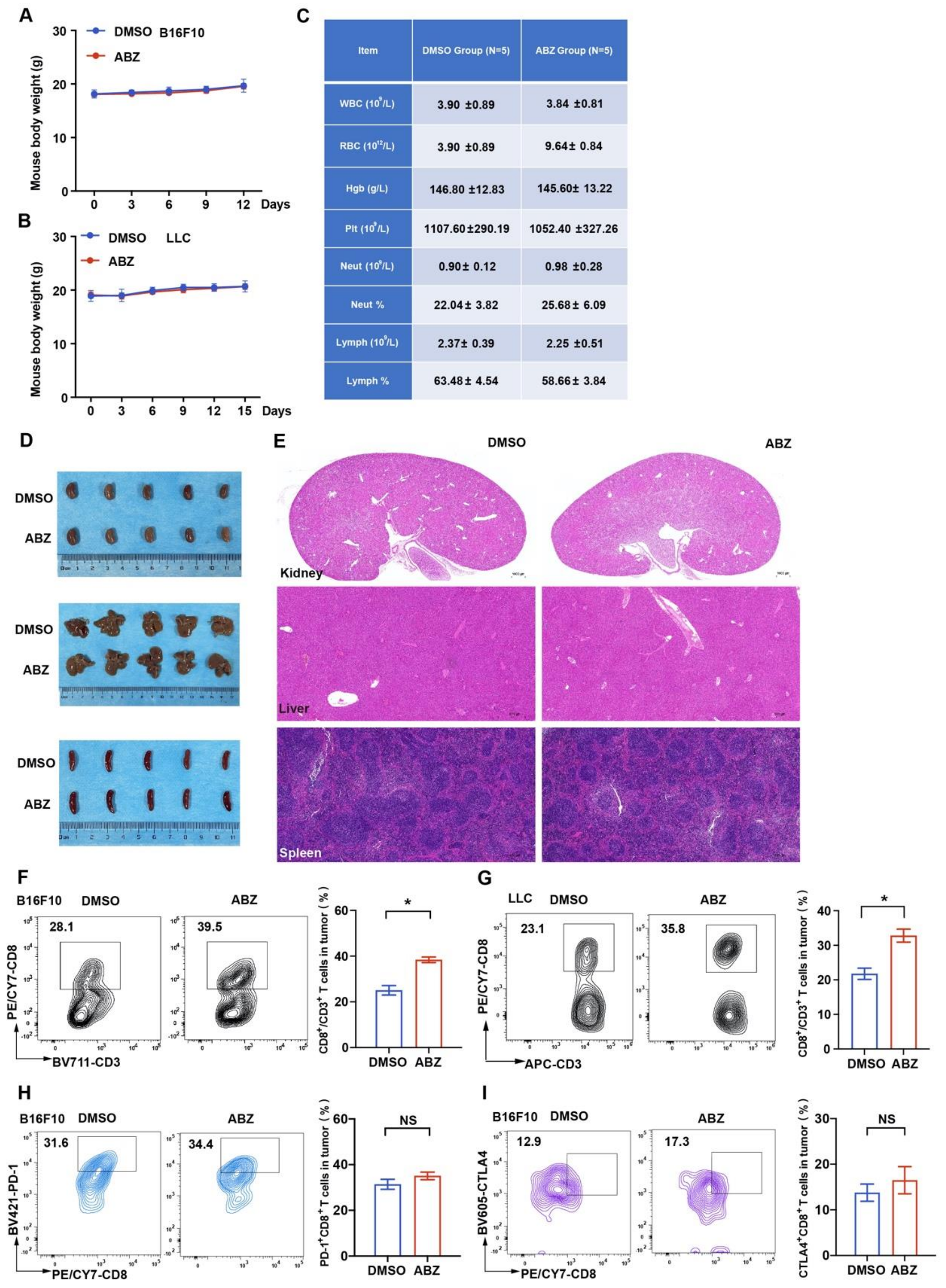

Item	DMSO Group (N=5)	ABZ Group (N=5)
WBC (10^9/L)	3.90 ±0.89	3.84 ±0.81
RBC (10^{12}/L)	3.90 ±0.89	9.64± 0.84
Hgb (g/L)	146.80 ±12.83	145.60± 13.22
Plt (10^9/L)	1107.60±290.19	1052.40 ±327.26
Neut (10^9/L)	0.90± 0.12	0.98 ±0.28
Neut %	22.04± 3.82	25.68± 6.09
Lymph (10^9/L)	2.37± 0.39	2.25 ±0.51
Lymph %	63.48± 4.54	58.66± 3.84

A-B. Mice weight was measured on the indicated different time points after C57BL/6 mice were implanted with 1×10^6 B16F10 (A) or 1×10^6 LLC (B) and received 50 mg/kg of ABZ treatment. C. The blood routine examination was detected on the day

of LLC tumors were harvested. D-E. The morphology (D) and organizational structure (E) of kidney, liver, and spleen of LLC mice model treated with ABZ or DMSO were observed by H&E staining after euthanizing the mice. F-G. Representative profiles and quantification of flow cytometry-based detection of the $CD8^+$ in $CD3^+$ TILs in B16F10 (F) and LLC (G) tumor mass from the different treatment groups (n=5 mice per group). Data represent mean±SD, *p<0.05. H-I. Representative profiles and quantification of flow cytometry-based detection of the $PD\text{-}1^+$ (H) and CTLA4 (I) in $CD8^+$ TILs in B16F10 tumor mass from the different treatment groups (n=5 mice per group). Data represent mean±SD, $^{NS}p>0.05$.

Supplementary Figure 3

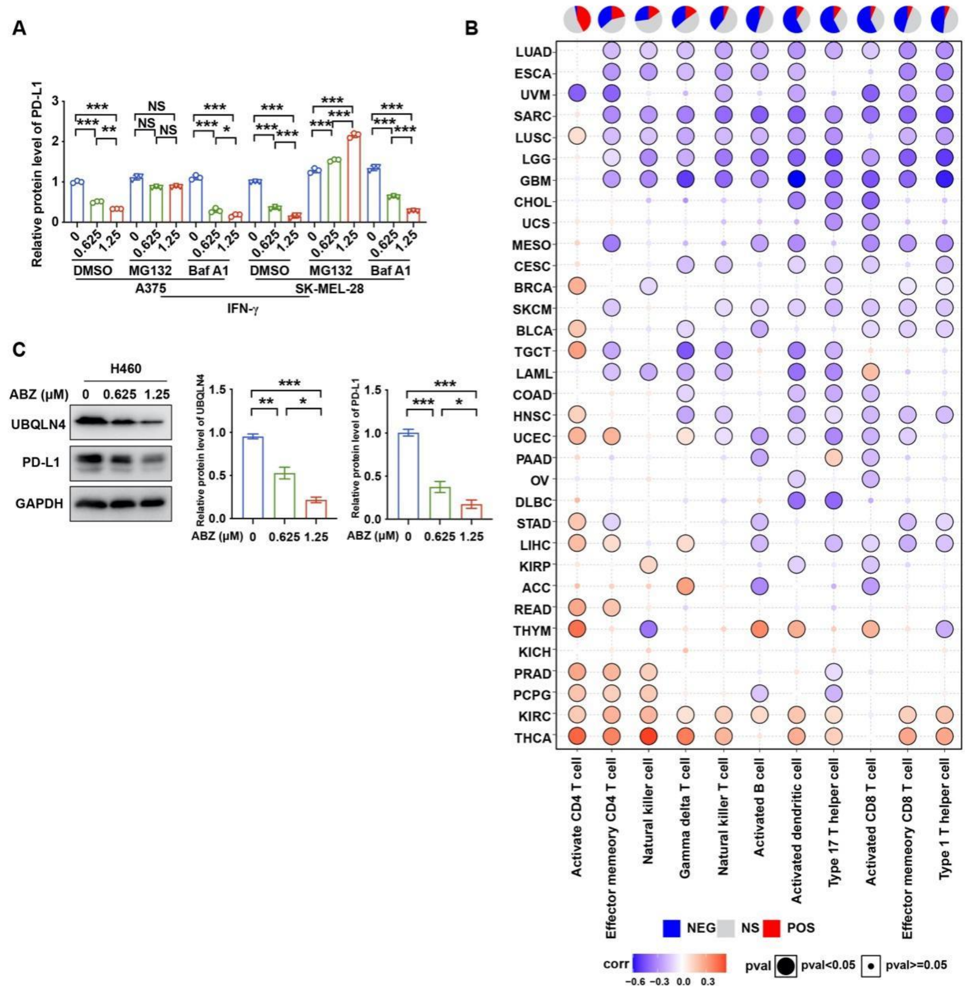

A. Quantitative analysis of PD-L1 protein expression in A375 and SK-MEL-28 cells, which were pretreated with Baf A1 (100 nM) and MG132 (10 μM) for 6 h then treated with increasing concentrations of ABZ (0.625 to 1.25 μM) and the PD-L1 protein level were detected by western blotting, each bar represents the mean±S.D of three independent experiments, $^{NS}p>0.05$, $*p<0.05$, $**p<0.01$, $***p<0.001$. B. Significantly correlation between UBQLN4 expression level and infiltration of the activated immune cell across 33 cancer types from TCGA. Pie charts summarized the proportion of cancer types with positive (Rs > 0 and pvalue < 0.05, in red), negative (Rs < 0 and pvalue < 0.05, in blue), or non-significant (in gray) correlation in 33

cancer types. Spearman's correlation test were performed, black border, *p < 0.05. C. Representative western blotting and quantitative analysis of UBQLN4 and PD-L1 protein levels in H460 cells treated with increasing concentrations of ABZ (0.625 to 1.25 μM) for 24 h, each bar represents the mean ±S.D of three independent experiments, *p<0.05, **p<0.01, ***p<0.001.

Supplementary Figure 4

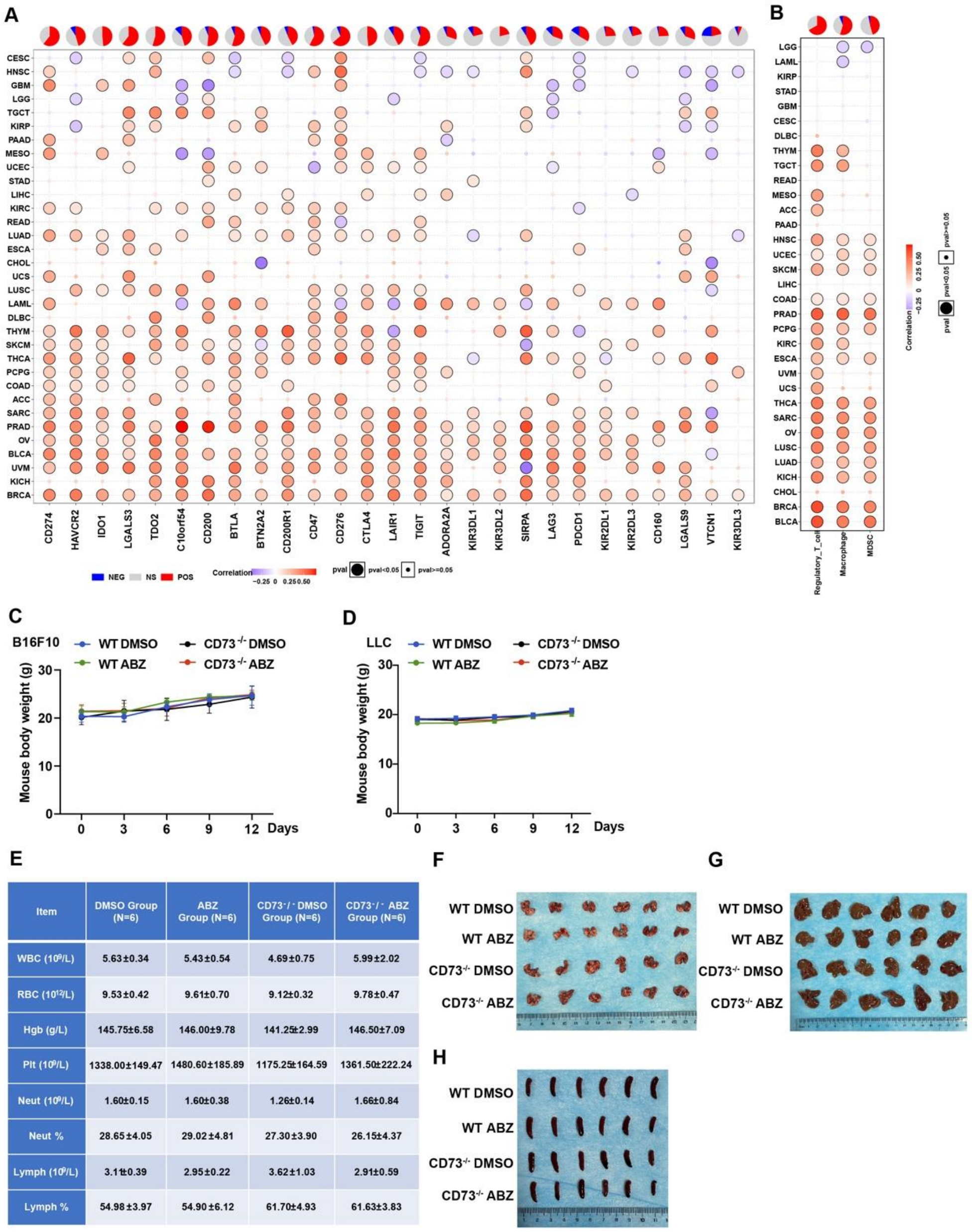

Item	DMSO Group (N=6)	ABZ Group (N=6)	CD73-/- DMSO Group (N=6)	CD73-/- ABZ Group (N=6)
WBC (10^9/L)	5.63±0.34	5.43±0.54	4.69±0.75	5.99±2.02
RBC (10^{12}/L)	9.53±0.42	9.61±0.70	9.12±0.32	9.78±0.47
Hgb (g/L)	145.75±6.58	146.00±9.78	141.25±2.99	146.50±7.09
Plt (10^9/L)	1338.00±149.47	1480.60±185.89	1175.25±164.59	1361.50±222.24
Neut (10^9/L)	1.60±0.15	1.60±0.38	1.26±0.14	1.66±0.84
Neut %	28.65±4.05	29.02±4.81	27.30±3.90	26.15±4.37
Lymph (10^9/L)	3.11±0.39	2.95±0.22	3.62±1.03	2.91±0.59
Lymph %	54.98±3.97	54.90±6.12	61.70±4.93	61.63±3.83

A-B. Significantly correlation for inhibitory immune checkpoints (A) or suppressive immune cell types (B) and CD73 expression level across 33 cancer types from TCGA. Spearman's correlation test were performed, black border, *p < 0.05. Pie charts

summarized the proportion of cancer types with positive (Rs > 0 and pvalue < 0.05, in red), negative (Rs < 0 and pvalue < 0.05, in blue), or non-significant (in gray) correlation in 33 cancer types. C-D. Mice weight was measured on the indicated different time points after CD73 WT and KO mice (n=6 mice per group) were implanted with 1×10^6 B16F10 (C) or 1×10^6 LLC (D) and received 50 mg/kg of ABZ treatment. E. The blood routine examination of CD73 WT and KO mice (n=6 mice per group) treated with ABZ or DMSO was detected on the day of LLC tumors were harvested. F-H. The morphology of lung (F), liver (G), and spleen (H) of CD73 WT and KO mice (n=6 mice per group) treated with ABZ or DMSO were observed after euthanizing the mice.

Supplementary Figure 5

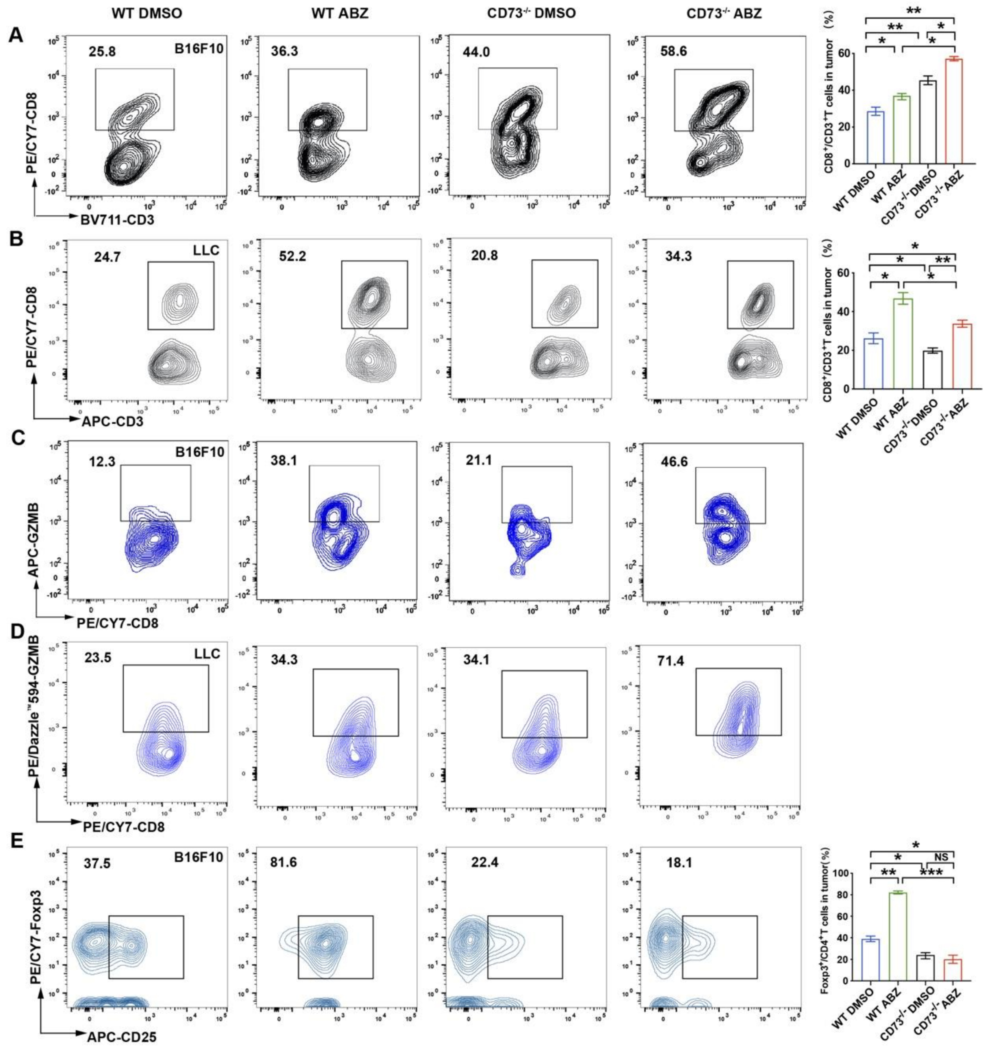

A-B. Representative profiles and quantification of flow cytometry-based detection of the CD8$^+$ in CD3$^+$ tumor infiltrates lymphocytes (TILs) in B16F10 (A) and LLC (B) tumor mass from the different treatment groups (n=5 mice per group). All data represent mean±SD, *p<0.05, **p<0.01. C-D. Representative profiles of flow cytometry-based detection of the GZMB$^+$ CD8$^+$ in CD3$^+$ TILs from B16F10 (C) and LLC (D) tumor mass. E. Representative profiles and quantification of flow cytometry-based detection of the Foxp3$^+$ in CD4$^+$ TILs in B16F10 tumor mass from the different treatment groups (n=5 mice per group). All data represent mean±SD, ^{NS}p>0.05, *p < 0.05, **p<0.01, ***p<0.001.

Supplementary Figure 6

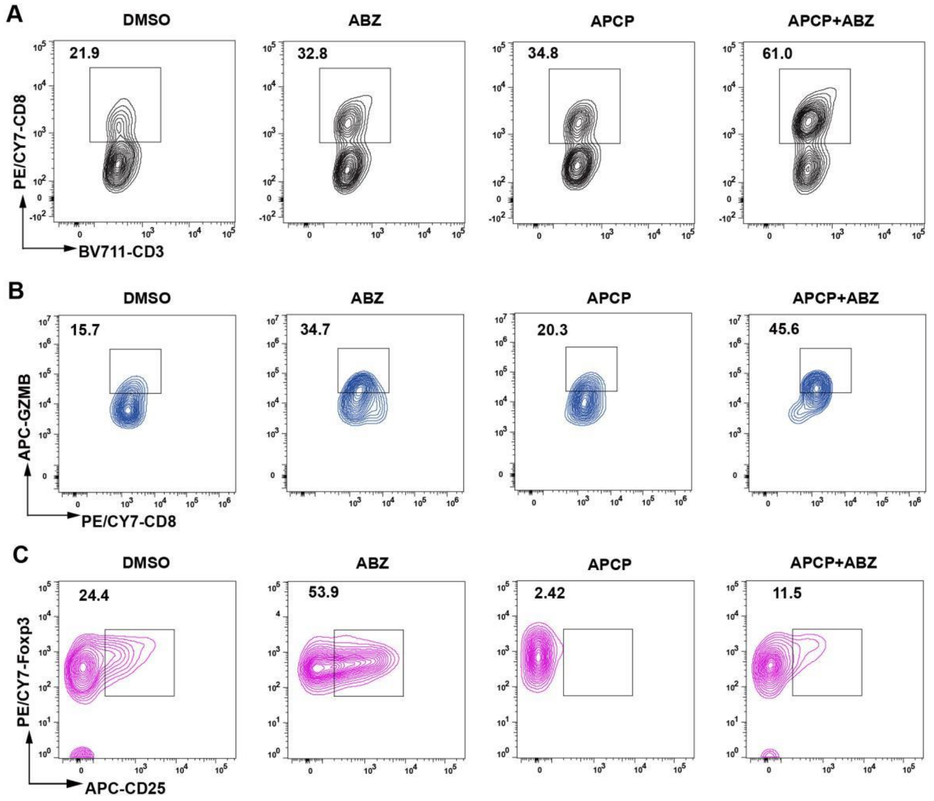

A. Representative profiles of flow cytometry-based detection of the CD8$^+$ in CD3$^+$ TILs in B16F10 tumor mass from the different treatment groups. B. Representative profiles of flow cytometry-based detection of the GZMB$^+$CD8$^+$ in CD3$^+$ TILs from the B16F10 tumor mass. C. Representative profiles of flow cytometry-based detection of the FOXP3+ in CD4+ TILs in B16F10 tumor mass from the different treatment groups.

Supplementary Figure 7

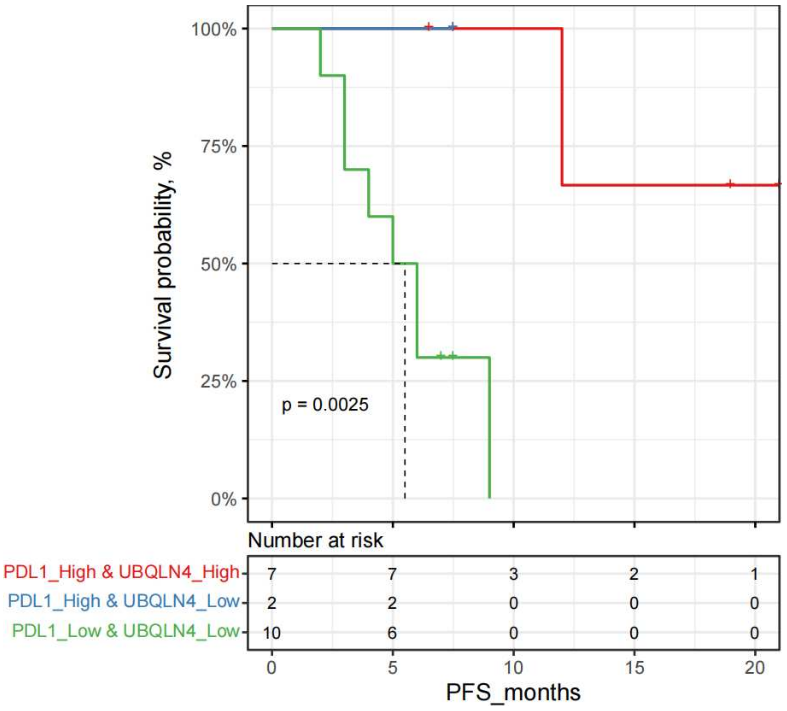

Melanoma patients with low UBQLN4 and low PD-L1 expression showed the worst efficacy of PD-1 mAb therapy.

Supplementary Table 1. Reagent or resource source identifier.

REAGENT or RESOURCE	SOURCE	IDENTIFIER
Antibodies		
Trustain fcX anti-mouse CD16/CD32	BioLegend	Cat# 101320
Zombie Aqua™ Fixable Viability Kit	BioLegend	Cat#423102
APC/Cy7 anti-mouse CD45	BioLegend	Cat#103116
Brilliant Violet 711 anti-mouse CD3	BioLegend	Cat#100241
APC anti-mouse CD3	BioLegend	Cat#100236
PerCP/Cyanine5.5 anti-mouse CD4	BioLegend	Cat#100434
PE/Cy7 anti-mouse CD8a	BioLegend	Cat#100722
APC anti-mouse GZMB	BioLegend	Cat#372204
PE/Dazzle™594 anti-mouse GZMB	BioLegend	Cat#372216
Brilliant Violet 605 anti-mouse CTLA4	BioLegend	Cat#369610
Brilliant Violet 421 anti-mouse PD1	BioLegend	Cat#135218
Brilliant Violet 421 anti-mouse CD274 (PD-L1)	BioLegend	Cat#124315
APC anti-mouse PD-L1	BioLegend	Cat#124312
APC anti-mouse CD25	Ebioscience	Cat#17-0251-82
PE/Cyanine7 anti-mouse FOXP3	Ebioscience	Cat#25-5773-82
PE anti-human CD274 (PD-L1)	BioLegend	Cat#329706
PE anti-human CD8	BioLegend	Cat#980902
Brilliant Violet 650™ anti-human CD279 (PD-1)	BioLegend	Cat#367429
APC/Cyanine7 anti-human CD3	BioLegend	Cat#300317
Human TruStain FcX	BioLegend	Cat#422302
Human anti-PD-L1-Rb	Abcam	Cat#ab213524<br>Cat#ab205921
PD-L1 (E1L3N®) XP® Rabbit mAb	Cell Signaling Technology	Cat#13684
PD-L1/CD274 Monoclonal Antibody	Proteintech	Cat#66248-1-Ig

UBQLN4 Antibody (A333, A1UP)	Santa Cruz Biotechnology	Cat#sc-136145
Ubiquitin Antibody (P4D1)	Santa Cruz Biotechnology	Cat#sc-8017
GAPDH Antibody (A-3)	Santa Cruz Biotechnology	Cat#sc137179
GAPDH Monoclonal Antibody	Proteintech	Cat#60004-1-Ig
HA-Tag antibody	Cell Signaling Technology	Cat#3724
His-Tag antibody	Cell Signaling Technology	Cat#12698
Peroxidase AffiniPure Goat Anti-Mouse IgG (H+L)	Jackson ImmunoResearch	Cat#115-035-146
Peroxidase AffiniPure Goat Anti-Rabbit IgG (H+L)	Jackson ImmunoResearch	Cat#111-035-144
BeyoECL Star	Beyotime Biotechnology	Cat#P0018AM
Chemicals, peptides, and recombinant proteins		
Albendazole (SKF-62979)	Selleck Chemicals	Cat#S1640
MG132	Selleck Chemicals	Cat#S2619
Bafilomycin A1 (Baf-A1)	Selleck Chemicals	Cat#S1413
Cycloheximide	MCE	Cat#HY-12320
Dimethyl sulfoxide (DMSO)	Sigma-Aldrich	Cat# D8418
Adenosine 5'-(α,β-methylene)diphosphate sodium salt(APCP)	Tocris	Cat# 3633
Recombinant Human IFN-gamma Protein	R&D Systems	Cat#285-IF

Isolation of peripheral blood lymphocytes	Tbdscience	Cat#LTS1077
CTSTM AIIM VTM SFM	Gibco	Cat#A3021002
Immuno Cult Human CD3/CD28/CD2 T cell activator	STEMCELL Technologies	Cat#10970
Recombinant Human IL-2	R&D Systems	Cat#202-1L-050
TRIzol™ Reagent	Invitrogen™	Cat#15596018
SuperScript III First-Strand	Invitrogen™	Cat#18080051
RIPA Lysis buffer	Beyotime Biotechnology	Cat#P0013B
Cell lysis buffer for Western and IP	Beyotime Biotechnology	Cat#P0013
Pierce™ BCA Protein Assay Reagent kit	Thermo Scientific™	Cat#23228; Cat# 23224
Nitrocellulose (NC) membranes	Merck KGaA	Cat#Z358657
PhosStop	Roche	Cat#4906845001
Cocktail	Roche	Cat#11836145001
Protein A/G magnetic beads	Bimake	Cat#B23201
Anti-HA magnetic beads	Bimake	Cat#B26201
RiboFECT CP Transfection Kit	RiboBio	Cat#C10511-05
Purified human Protein His-PD-L1	Sino Biological	Cat#10084-H08H
Puromycin (CL13900) 2HCl	Selleck Chemicals	Cat#S7417
Polybrene	Solarbio life sciences	Cat#H8761
Turbofect Transfection ragent	Thermo Scientific	Cat#R0531
Experimental models: Cell lines		

A375	ATCC	Cat#CRL-1619
SK-MEL-28	ATCC	Cat#HTB-72
A549	In our lab	N/A
H460	In our lab	N/A
B16F10	ATCC	Cat#CRL-6475
LLC	ATCC	Cat#CRL-1642
HEK293T	In our lab	N/A
Oligonucleotides		
Control siRNA1	GenePharma	N/A
5’-GAAATGTACTGCGCGTGGAGAC-3’		
Ubqln4-siRNA1	GenePharma	N/A
5’-GGTCAGGGATGTTCAATAG-3’		
Ubqln4-siRNA2	GenePharma	N/A
5’-CAATAACCCTGAACTCATG-3’		
Human sgUBQLN4#1	Shanghai	N/A
Forward：5’-caccgtggggtccgggatatcccaa-3’	sangon biotech	
Human sgUBQLN4#1	Shanghai	N/A
Reverse：5’-aaacttgggatatcccggaccccac-3’	sangon biotech	
Human sgUBQLN4#2	Shanghai	N/A
Forward：5’-caccgctgtactacctccttgaccg-3’	sangon biotech	
Human sgUBQLN4#2	Shanghai	N/A
Reverse：5’-aaaccggtcaaggaggtagtacagc-3’	sangon biotech	
Human PD-L1 primer	Shanghai	N/A
Forward:5’-TATGGTGGTGCCGACTACAA-3’	sangon biotech	
Human PD-L1 primer	Shanghai	N/A
Reverse:5’-TGCTTGTCCAGATGACTTCG-3’	sangon biotech	
GAPDH primer	Shanghai	N/A
Forward:5’-CATGAGAAGTATGACAACAGCCT-3’	sangon biotech	
GAPDH primer	Shanghai	N/A

Reverse:5'-AGTCCTTCCACGATACCAAAGT-3'	sangon biotech	
Experimental models: Organisms/strains		
C57BL/6J	Shanghai SLAC	N/A
CD73$^{-/-}$ C57BL/6J	Shanghai SLAC	N/A
Software and Algorithms		
GraphPad Prism 5.0	GraphPad Software, Inc	https://www.graphpad.com/
CRISPR design	N/A	http://crispr.mit.edu/
FlowJo 10.0	TreeStar	N/A
Image Lab	Bio-Rad	https://www.bio-rad.com
Prism 8	GraphPad	https://www.graphpad.com/

Supplementary Table 2. Clinicopathologic characteristics of anti-PD-1 monotherapy cohorts.

PID	Gender	Age	Subtype	Site	Tumor lesion	TNM stage	Stage	PS score	PFS months	Response	Metastatic Lesion
1	Female	46	Acral	metastatic	Left heel	T2bN2cM0	Stage III	1	4	NR	Popliteal lymph nodes
2	Male	71	Nodular	metastatic	Left leg	T3bN3M1a	Stage IV	1	6	NR	Inguinal lymph nodes, left leg skin
3	Female	37	Acral	primary	Left heel	T1aN1aM0	Stage IIIA	1	7.5+	R	Popliteal lymph node
4	Female	58	Acral	primary	Left heel	T4bN1aM0	Stage IIIB	1	7.5+	R	Inguinal lymph node
5	Male	68	Acral	primary	Left heel	T3bN1aM0	Stage IIIA	1	7.5+	R	Inguinal lymph node
6	Female	11	Nodular	primary	Left leg	T4aN2bM0	Stage IIIB	1	7.5+	R	Inguinal lymph nodes
7	Female	68	Acral	primary	Right heel	T3bN2bM1c	Stage IV	1	6.5+	R	Inguinal lymph nodes, Lung
8	Female	64	Acral	primary	Right heel	T4bN1aM0	Stage IIIB	1	7+	R	Inguinal lymph node
9	Male	50	Nodular, with ulceration	primary	Right heel	T4bN0M0	Stage IIC	1	7.5+	R	None
10	Female	71	Acral	metastatic	Right foot	T2N1M1C	Stage IV	1	3	NR	Lung, brain
11	Female	35	Nodular	primary	Left back	TxN2M1	Stage IV	1	12	R	Liver, bone, brain
12	Female	56	Acral	primary	Right heel	T1bN1aM0	Stage IIIA	1	7.5+	R	Inguinal lymph node
13	Female	70	Nodular	primary	Right foot	T4bN2bM1a	Stage IV	1	6	NR	Neck and inguinal lymph nodes
14	Male	46	Nodular	primary	Scalp	T4bN0M1	Stage IV	1	5	NR	Lung
15	Female	64	Superficial spreadin	metastatic	Left foot	T2bN0M1a	Stage IV	1	9	R	Scalp, gastric area
16	Female	64	Nodular	metastatic	Right leg	TxN2M0	Stage IIIA	1	2	NR	Inguinal lymph nodes
17	Female	56	Acral	primary	Left thumb	T4bN3cM1c	Stage IV	1	19+	R	Inguinal and retroperitoneal lymph nodes
18	Female	38	Nodular	primary	Right index f inger	T3aN2bM0	Stage IIIB	1	21+	R	Axillary, subclavian and elbow fossa lymph nodes
19	Female	75	Nodular	metastatic	Right finger	T3bN3M0	Stage IIIC	1	3	NR	Axillary and chest wall lymph nodes

Patients were stratified into response groups based on RECIST 1.1 criteria. TNM stage based on the 8th Edition Melanoma Stage Classification. PFS, progression-free survival; R, response; NR, non-response.

Supplementary Table 3. The list of 30 candidate proteins detected by MS.

Protein ID	Avg. Mass	Description
35	53652	Vimentin sapiens GN=VIM
48	33296	Prohibitin-2 sapiens GN=PHB2
47	28994	Emerin sapiens GN=EMD
118	36984	Serine/threonine-protein phosphatase PP1-gamma catalytic subunit sapiens GN=PPP1CC
70	38659	Putative annexin A2-like protein sapiens GN=ANXA2P2
69	38604	Annexin A2 sapiens GN=ANXA2
142	39233	Pyruvate dehydrogenase E1 component subunit beta mitochondrial sapiens GN=PDHB
194	226530	Myosin-9 sapiens GN=MYH9
2741	58887	Solute carrier family 35 member F5 sapiens GN=SLC35F5
3252	171197	Transient receptor potential cation channel subfamily M member 2 sapiens GN=TRPM2
61	34930	Leucine-rich repeat-containing protein 59 sapiens GN=LRRC59
234	55537	COP9 signalosome complex subunit 1 sapiens GN=GPS1
236	53445	Serine/threonine-protein kinase ULK3 sapiens GN=ULK3
202	63853	Ubiquilin-4 sapiens GN=UBQLN4
4605	41443	Circadian-associated transcriptional repressor sapiens GN=CIART
4612	52186	General transcription factor IIH subunit 4 sapiens GN=GTF2H4
4629	64685	Myosin light chain kinase 2 skeletal/cardiac muscle sapiens GN=MYLK2
1691	125830	Angiopoietin-1 receptor sapiens GN=TEK
2992	138109	Filamin-A-interacting protein 1 sapiens GN=FILIP1
4967	179402	Thrombospondin type-1 domain-containing protein 7B sapiens GN=THSD7B
5417	316911	Protein PRRC2C sapiens GN=PRRC2C
196	27077	Clathrin light chain A sapiens GN=CLTA
148	307550	A-kinase anchor protein 13 sapiens GN=AKAP13
4601	288984	Spectrin beta chain non-erythrocytic 4 sapiens GN=SPTBN4
224	138386	Sperm-specific antigen 2 sapiens GN=SSFA2
4634	51544	GDNF family receptor alpha-2 sapiens GN=GFRA2
4654	66959	CREB-regulated transcription coactivator 3 sapiens GN=CRTC3
4710	103702	Calcium homeostasis endoplasmic reticulum protein sapiens GN=CHERP
4713	106834	Centromere protein C sapiens GN=CENPC
4846	140467	Contactin-associated protein-like 3B sapiens GN=CNTNAP3B
