# Supplementary material for: Albendazole induces immunotherapy response by facilitating ubiquitin-mediated PD-L1 degradation
Source: J Immunother Cancer. 2022 May 16;10(5):e003819. doi: 10.1136/jitc-2021-003819 (PMC9115032; doi:10.1136/jitc-2021-003819)
Supplement: Supplementary data [file jitc-2021-003819supp001.pdf]

Graphical Abstract

Albendazole induces immunotherapy response by facilitating ubiquitin-mediated PD-L1 degradation

Lin Zhu, Xinwei Kuang, Guanxiong Zhang, Long Liang, Dandan Liu, Bin Hu, Zuozhong Xie, Hui Li, Hong Liu, Mao Ye, Xiang Chen, Jing Liu

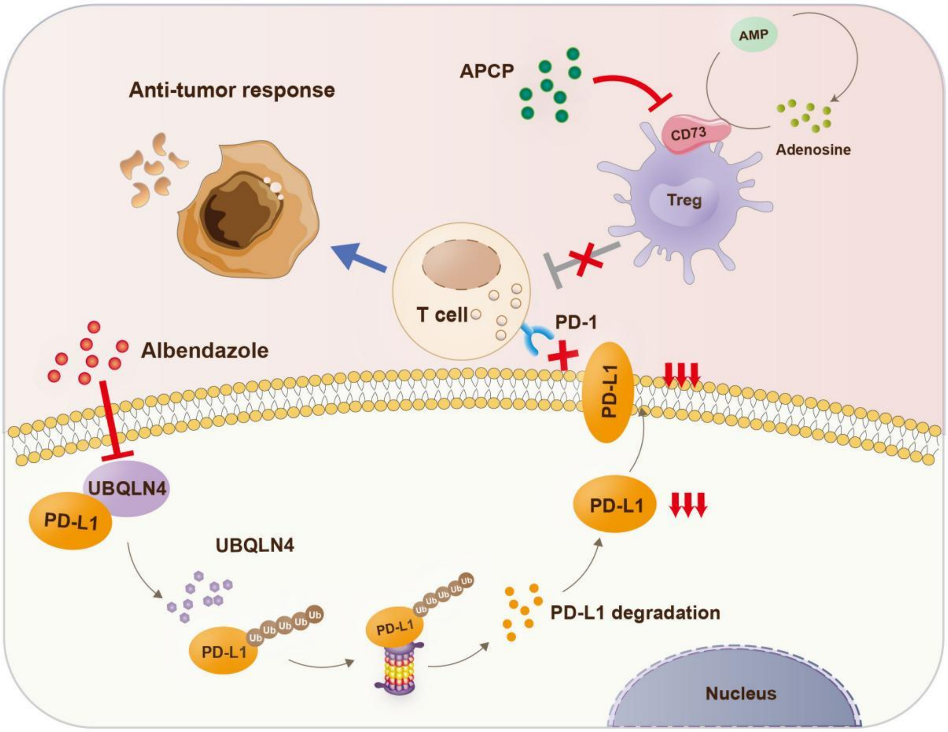

Our findings revealed a novel role of ABZ in anti-tumor immunity by suppressing the UBQLN4-mediated stabilization of PD-L1 protein and inducing ubiquitin-mediated PD-L1 protein degradation, thereby provided a potential predictor kit (UBQLN4/PD-L1) for assessing the efficacy of anti-PD-1 therapy in clinic and a novel therapeutic strategy by combination treatment of ABZ and CD73 blockade.
